# Multidimensional Infrared Spectroscopy as a Bioanalytical Technique

**DOI:** 10.1021/acs.chemrev.6c00244

**Published:** 2026-07-16

**Authors:** Sander Woutersen, Neil T. Hunt

**Affiliations:** † Van ’t Hoff Institute for Molecular Sciences, University of Amsterdam, 1098XH Amsterdam, The Netherlands; ‡ Department of Chemistry and York Biomedical Research Institute, 8748 University of York, York YO10 5DD, U.K.

## Abstract

Since its inception at the end of the last century, 2D-IR spectroscopy has undergone continuous development into an established method for studying the structure, dynamics and intermolecular interactions of biomolecules. Thanks to parallel advances in lasers and related technology, 2D-IR data sets can now be acquired in minutes, using minimal sample quantities and under physiologically relevant conditions. This progress has opened the door for 2D-IR spectroscopy to play a role as an analytical tool, making measurements that characterize samples and inform decisions in arenas ranging from drug design to biomedical diagnostics. Here, we briefly review the pioneering developments that have brought about this scenario before discussing the current state of the art in analytical applications of 2D-IR spectroscopy. We consider the implementation of 2D-IR spectroscopy to measure label free protein structure, biomolecular interactions and the use of 2D-IR to interrogate complex mixtures and biological tissues. We include strategies that have extended the reach of 2D-IR spectroscopy through advances in measurement sensitivity and data quality and highlight the potential impact that emerging methods such as machine learning could have on the development of 2D-IR. Finally, we look ahead to the challenges and opportunities facing 2D-IR on its path toward a robust and widely used bioanalytical tool.

## Graphical abstract

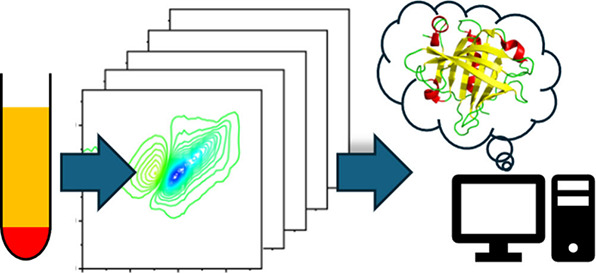

## 1 Introduction

First reported in 1998,^1^ ultrafast two-dimensional infrared (2D-IR) spectroscopy has made considerable advances over the intervening years. The origins of the technique are similar to those of multidimensional NMR spectroscopy, in which a complex spectrum is spread over a second frequency axis, giving greater detail and insight than can be achieved using one dimension.^2^ In the case of 2D-IR, the basis of the method lies in IR_pump_-IR_probe_ spectroscopy, where an infrared laser pulse, the pump, is used to excite a molecular vibration, before a second, probe, pulse is used to measure the state of the system some time (waiting time, *T*_w_) later. 2D-IR spectroscopy differs from standard IR_pump_-IR_probe_ methods through frequency resolution of the pump process so that a 2D-IR spectrum becomes a correlation map of pump (excitation) frequency versus probe (detection) frequency.^2^

The ability to observe the infrared spectral response of a molecule, which arises from vibrational modes of functional groups, over two frequency dimensions in a time-resolved manner has enabled many new insights. These include direct visualization of the couplings between molecular vibrations, which are manifest as off-diagonal peaks in the 2D-IR spectrum.^2^ The relationship between coupling strengths and the proximity and connectivity of functional groups can lead to direct structural information. The evolution of the spectrum with *T*_w_ adds dynamic information ranging from vibrational relaxation pathways to energy transfer routes between modes.^3–5^ Lineshapes and widths of IR absorption bands carry significant molecular information and extending the measurement to 2D has enabled contributions from homogeneous and inhomogeneous line broadening mechanisms to be separated, while the molecular dynamics that give rise to the effects can also be uncovered.^6,7^

The development of 2D-IR methods using a range of molecular systems has contributed to what is now a well-established suite of spectroscopic tools.^2^ In parallel, advances in technical aspects of the measurement and the equipment used to collect 2D-IR spectra have also occurred, such that experiments and data sets that represented the peak of technical achievement 20 years ago can now be acquired in minutes or even faster and with significant improvements in signal-to-noise ratio and detection sensitivity.^8^

This situation sets the stage for our review, which focuses on analytical applications of 2D-IR spectroscopy. Here, we define analytical applications as ones in which 2D-IR has been used to make a measurement that informs progress toward another scientific goal.^9^ This is a deliberately broad definition as, in principle, the applicability of 2D-IR has the potential to match that of its ancestors IR absorption and NMR spectroscopies, which are now ubiquitous in (bio)­chemical analysis laboratories. Specific examples might include providing information on structure or ligand binding that guides a drug design campaign, or sample categorization that enables a biomedical assessment to be made. The ability to write such a review reflects the evolution of 2D-IR spectroscopy and its implementation from a highly advanced laser spectroscopy technique, where the focus was on obtaining a single time-resolved data set, to one where near turnkey laser systems and commercial one-box spectrometers have allowed 2D-IR to interrogate a molecular problem or to compare a number of samples in the manner of a screening experiment. While 2D-IR spectroscopy still requires considerable technical expertise and expensive equipment, the direction of travel toward a method that can sit alongside established analytical spectroscopies will become clear. We will review progress in such applications, which focus largely on the last 5–7 years, but to do so we will need to highlight the considerable body of earlier work that brought the field to its current state. One area of 2D-IR research that lies beyond our scope however will be the large body of work relating to the use of site-specific probes and labeled proteins. These have contributed considerable insights, and will be reviewed elsewhere in this special collection, but the complexities of sample preparation involved mean that we do not view them as being readily applicable in an analytical context.

## 2 2D-IR Method and Information Content

The method for measuring 2D-IR spectra has been described in detail elsewhere and reviewed on several occasions.^2,10–17^ We will not recapitulate this here, but seek to give an overview of the types of spectral information that are available from 2D-IR spectra, and particularly those which contribute to analytical applications of 2D-IR spectroscopy.

### 2.1 General Appearance

A 2D-IR spectrum arises from the coincidence in the sample of three ultrashort infrared pulses (Figure 1(a)) that are tuned to be resonant with vibrational modes of a molecule (Figure 1(b)). The first two laser pulses lead to the pumping or excitation of a vibrational mode of interest while the third is used as a time delayed probe pulse and also acts as a local oscillator for the heterodyne-detected measurement.^2^ The two frequency dimensions of the 2D-IR spectrum arise from measuring the probe pulse (which by this stage is actually a colinear combination of the 2D-IR signal and residual probe light) in a spectrometer as a function of the time delay between the two pump pulses (τ), for a fixed *T*_w_. Thus, the spectrometer provides the probe axis of the spectrum directly, while Fourier transformation of the signal as a function of τ yields the pump frequency axis (Figure 1(c)). The sample is most often a solution held between two IR-transmissive windows that are separated by a spacer that defines the path length for the measurement, though, increasingly other sample types and measurement geometries are being employed.^18–23^

**Figure 1:**
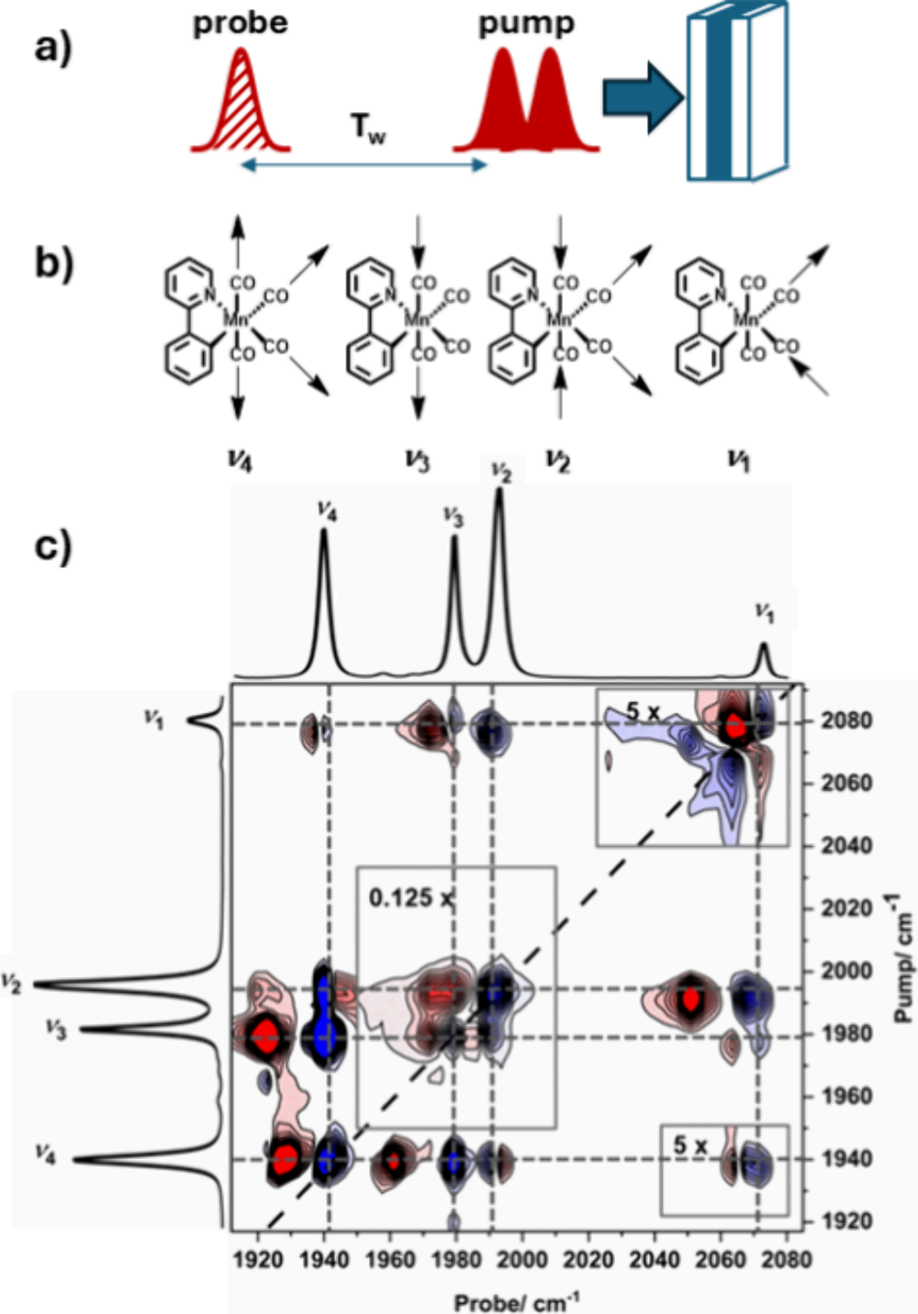
a) Schematic diagram of the IR pulse sequence used to generate a 2D-IR spectrum. b) and c) show an example spectrum in which the four carbonyl ligand stretching vibrational modes of [Mn­(ppy)­(CO)_4_] are measured. Panel b) describes the vibrational modes, while c) shows the 2D-IR spectrum. Panels b) and c) are reproduced from ref 24. Copyright 2024 American Chemical Society.

### 2.2 The Spectrum Diagonal

In the manner described, a 2D-IR spectrum is produced that has the general appearance of that in Figure 1(c) and comprises a plot of pump vs probe frequencies. This example spectrum shows the carbonyl ligand stretching modes of the precatalyst molecule Mn­(ppy)­(CO)_4_ [**1**] in heptane solution (Figure 1(c)) and allows us to examine the key features.^24^ Molecule **1** features four CO ligands that give rise to the same number of ν_CO_ ligand stretching vibrations near 2000 cm^–1^ (Figure 1(b)). In the 2D-IR spectrum, peaks due to the fundamental transitions (*v* = 0–1) of each of the ν_CO_ modes appear on the spectrum diagonal (where pump frequency equals probe frequency). Thus, the diagonal of a 2D-IR spectrum strongly resembles the IR absorption spectrum of a sample, though with certain important differences: As the 2D-IR signal arises from a third order nonlinear optical process, the signal intensity shows a quadratic dependence on the molar extinction coefficient (fourth power of the transition dipole moment) of the vibrational mode, rather than the linear dependence exhibited in absorption spectroscopy. This leads to peaks on the 2D-IR diagonal having a slightly narrower line width than is observed in absorption spectroscopy. In samples with multiple overlapping bands, this can lead to improved peak resolution as compared to FT-IR spectra. Despite the nonlinear nature of the 2D-IR response, it is important to note that the size of the peaks due to a particular molecule scale linearly with concentration (assuming negligible pump depletion), which is important for analytical applications.

The nonlinear dependence of 2D-IR signal size on the extinction coefficient of a mode can also lead to crucial differences in the way that solvent modes contribute to 2D-IR and absorption spectra. A consistent problem for FT-IR spectra arises from the dominance of solvent-derived bands, even in regions of the spectrum where no fundamental vibrational transitions occur. This is because the numerical dominance of the solvent molecules causes the absorbance of even weakly absorbing solvent bands, such as from overtones or combination transitions, to overwhelm fundamental modes of a low concentration solute. By contrast, a 2D-IR spectrum is dominated by modes with larger extinction coefficients, so that the solute bands dominate the response and the 2D-IR spectrum diagonal is largely ‘solvent background free’.^25^ An excellent example of this is seen in studies of the [NiFe] hydrogenase enzymes (Figure 2).^26^ These enzymes, which catalyze the reversible activation of molecular hydrogen, do so via an active site containing CO and CN ligands coordinated to an Fe atom in a bimetallic (NiFe) cluster. These anaerobic enzymes can only be prepared at mM concentrations leading to very small (few mOD, Figure 2(a)) absorbances, even for the strongly absorbing ν_CO_ modes.^26–29^ Indeed, the FT-IR spectrum in the 2000 cm^–1^ region is dominated by the bend-libration combination band of water (Figure 2(a)) such that the carbonyl and cyano stretching mode bands of the enzyme are virtually invisible (blue arrows).^26^ Difference absorption spectroscopy, subtracting the solvent response, can be used to reveal these bands more clearly (Figure 2(b), top panel, black line), but the signal-to-noise ratio is still poor. In contrast, a 2D-IR spectrum of the same sample (Figure 2(b)) shows no contribution from the solvent combination band while peaks due to the ν_CO_ and ν_CN_ modes are not only clear, but the quality of the spectrum is such that multiple peaks from several redox states of the enzyme are clearly visible.^26^ An additional piece of spectroscopic information relating to the diagonal peaks arises from the positive peaks that appear shifted to lower probe frequency relative to each diagonal peak. These peaks originate from the *v* = 1–2 transition of the mode. Thus, the probe frequency separation of these peaks provides a direct measurement of the anharmonicity of the mode.^26,27^

**Figure 2:**
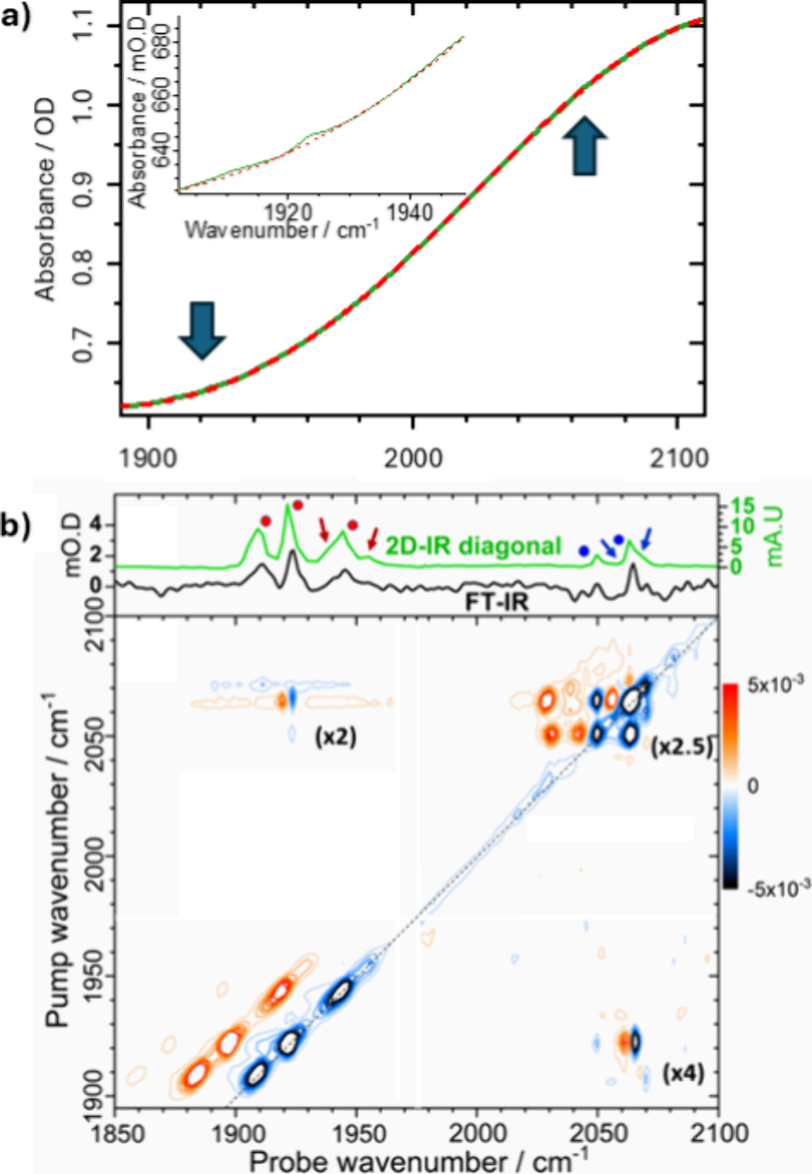
a) FTIR spectrum and b) 2D-IR spectrum of a [NiFe] hydrogenase enzyme, which features an active site containing an Fe­(CO)­(CN)_2_ moiety. Under the conditions studied, the stretching vibrational modes of the carbonyl and cyano ligands are barely visible in FTIR measurements (a) where the spectrum is dominated by a combination band of H_2_O. By contrast the 2D-IR spectrum shows these modes clearly, with no apparent contribution from the solvent band. Reproduced from ref 26. Available under a CC-BY-3.0 license. Copyright Neil T. Hunt, Alison Parkin.

In addition to information relating to fundamental vibrational modes of the sample, the 2D-IR diagonal can be used to extract dynamic information. For example, by controlling the relative polarization directions of the pump and probe pulses, vibrational lifetimes and molecular reorientation times can be obtained via the change in amplitude of the diagonal peaks as a function of *T*_w_.^2^

In the case of inhomogeneously broadened lineshapes, which are caused by the solvent or molecular environment exerting differing influences on the molecules in the sample that give rise to the spectrum, the underlying dynamics of the system causing the broadening can be extracted by observing the change in diagonal 2D line shape as a function of *T*_w_, referred to as spectral diffusion.^6,30^ While both vibrational lifetime and spectral diffusion can be applied analytically, they will not be the focus of the studies that we discuss below and so we refer the interested reader to other review articles for more information.^2,10–17^

### 2.3 The off-Diagonal Region of the Spectrum

Moving to the off-diagonal region of the 2D-IR spectrum of our example molecule **1** (Figure 1(c)) we can see a large number of peaks.^24^ In contrast to the diagonal peaks, which have identical pump and probe frequencies, off-diagonal peaks have different values of pump and probe frequency. The off-diagonal peaks in Figure 1(c) occur in pairs containing a blue (negative) peak, which links two of the diagonal peaks, and a red, positive, peak. These are shown in the figure by dashed guidelines. In the example spectrum shown in Figure 1(c), obtained at a near-zero value of *T*_w_, the presence of these pairs of peaks provide evidence of vibrational coupling between the ν_CO_ modes of molecule **1**, i.e. that excitation of one mode leads to a change in frequency of the other.^24^ The frequency separation of the positive–negative peak pair along the probe frequency axis of the spectrum indicates the off-diagonal anharmonicity, which is reflective of the coupling strength of the two modes.^2,24,31^ The polarization sensitivity of the off-diagonal peaks can also be used to extract angles between the transition dipole moment directions of the coupled modes.^31^

Observing the off-diagonal region of the 2D-IR spectrum as a function of *T*_w_ leads to an evolution of the peaks as a result of vibrational relaxation. This can manifest as an increase in the amplitude of the off-diagonal peaks relative to the diagonal peaks as energy is redistributed between the *v* = 1 levels of the vibrational modes. In some cases, where line widths are small, the appearance of additional peaks due to energy transfer is resolvable.^24,31^

A considerable amount of detailed information can be extracted from the off-diagonal region of a 2D-IR spectrum. From an analytical perspective, these features have several uses, many of which stem from treating the 2D peak pattern arising from a particular molecule simply as a spectral fingerprint.^32^ Such an approach simplifies the analysis of data considerably and may be sufficient for the application. When utilizing such a 2D-IR pattern, it is important to recall that the spectral information encoded in the pattern is significantly more detailed than that obtained in 1D given that it contains information on intermode couplings. Such patterns or fingerprints can be used to identify species, but also to unravel spectra from complex mixtures where diagonal peaks, or peaks in the absorption spectrum, overlap.^32^ In the case of the spectra of mixtures, the 2D response is additive, as it would be in an absorption spectrum. If measuring the spectrum as a function of *T*_w_, then the evolution of these spectral patterns due to relaxation creates an additional layer of information that could be used to separate and identify molecular contributions to a mixture. Thus, the 2D-IR off-diagonal peaks of a molecule offer a multifaceted spectral fingerprint for recognition.

## 3 Proteins and Biomolecules

### 3.1 The 2D-IR response of proteins

As we shall see, the development of 2D-IR spectroscopy has included a significant focus on measurements of biomolecules, and proteins in particular. This is due in large part to the sensitive nature of the amide I infrared band of proteins and peptides to secondary structure. Combining this sensitivity with the ability to spread the response over a second dimension and suppress solvent bands creates the opportunity for 2D-IR to deliver structural information in the physiologically relevant solution phase.^1,17,33–39^

The structure sensitivity of the amide I band, which is largely attributable to the C=O stretching motion of the peptide link, arises from its intimate relationship to the backbone configuration of a protein. A major influence on the C=O stretching frequency arises from through bond and through space coupling to neighboring residues. When peptides are folded into 3D secondary structure elements, the coupling between C=O oscillators extends across the macroscopic structure such that, rather than describing the amide I as a C=O stretching vibration, a delocalized description is more accurate, leading to characteristic vibrational modes of secondary structural features such as the α-helix or β-sheet (Figure 3).^1,17,33–39^ Other contributions to couplings occur from inter-residue hydrogen bonding within the secondary structural elements, while the degree of disorder or dynamic motion can affect the extent of all couplings. Finally, the local environment in which a residue is located, including solvent access, will influence the local electric field experienced by the C=O bond and so its stretching frequency and interactions with other modes.

**Figure 3:**
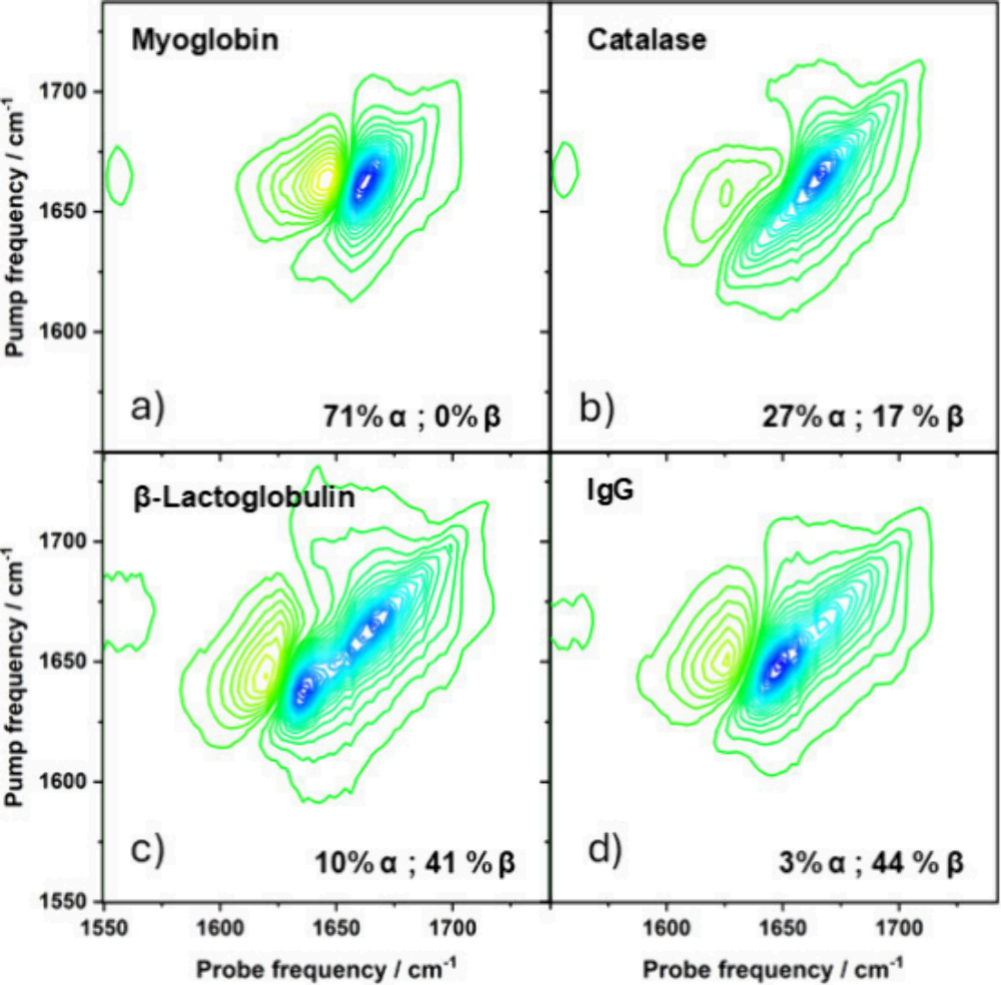
Example 2D-IR spectra of proteins featuring differing secondary structure compositions. In each case the contributions of α-helix and β-sheet to the structure are shown in the lower right corner of the spectrum. The degree of β-sheet increases from panel a) to d). Adapted from ref 40. Available under a CC-BY-3.0 license. Copyright Neil T. Hunt.

As the structure of a protein is intimately connected to its function, the 3D folded arrangement of the backbone is unique, but from an IR spectroscopy perspective its amide I band can be represented as a largely additive combination of signals from the individual secondary structure components (Figure 3), making basic analysis tractable.^41^ In comparison to linear spectroscopies, the ability of the 2D-IR response to act as a fingerprint of the structure is enhanced by the increased diagonal peak resolution and the presence of off-diagonal features due to coupling. In the former case, the narrowing of the diagonal peak widths makes it significantly easier to resolve contributions from e.g. α-helix and β-sheet into individual peaks, whereas in IR absorption spectroscopy the contributions are broader and overlap to the point where complex and often ill-defined fitting approaches are required to extract information. While the off-diagonal contributions to protein 2D-IR spectra (Figure 3) are less obvious than those in Figure 1(c), they remain valuable. In the case of proteins, the off-diagonal region tends to contribute to the overall pattern or shape of the peak rather than being present as a distinct spectral element. This is no less useful in terms of identifying structural features, unravelling mixtures or observing structure change, however.^42–45^

In the review that follows we will highlight the use of the amide I band in 2D as a tool for observing secondary structure variation and for gathering information about the content of complex proteinaceous mixtures. In doing so, it is important to recognize the focus on the use of label free protein 2D-IR spectroscopy. The examples that we will discuss focus on the amide I band of the whole protein and treat this band as a fingerprint of the structure. This approach is distinct from studies that implant specific vibrational labels or isotopically enrich portions of the structure to generate spatially localized information.^46–52^ The latter approaches employing labeling are extremely valuable and offer complementary insight to the label free method, but we focus our attention on the latter because, from an analytical perspective, it is desirable to work on as-isolated proteins without the need for time-consuming, expensive and potentially disruptive labeling strategies, while recognizing that this speed and efficiency comes at the expense of local insight.

### 3.2 Measuring Protein Structure

The use of 2D-IR spectroscopy for determination of absolute protein structures has been motivated by the potential for 2D-IR to provide a powerful complementary viewpoint to methods such as cryo-electron microscopy and X-ray crystallography. These yield extremely high-resolution structures in the solid phase. In contrast, 2D-IR offers physiological temperature, solution phase measurements, while what it lacks in atomistic detail is balanced by the ability to make time-resolved measurements that offer the potential to access dynamic structures.

When focusing on the label-free approach discussed above, the overarching challenge has been to decode the link between the 2D spectral signature and the 3D structure of a protein. The general relationship between the form of the amide I absorption band, in 1D, and secondary structure had long been known,^39^ but 2D-IR spectroscopy offered the ability to measure the underlying vibrational couplings directly. As such, considerable activity was dedicated to measuring the impact of the conformation, and hydrogen bonding, of small peptides on the coupling between C=O units.^1,17,35,39,43–45,53–65^ These studies progressed toward small model systems with well-defined secondary structures, for example elucidating the 2D signature of an antiparallel β-sheet and showing that the intensity ratio of the two characteristic bands gives an indication of the size of the sheet.^64^ The importance of the off-diagonal contribution to the spectrum became clear through these studies as coupling between the two main β-sheet modes and the involvement of disordered contributions at intermediate frequencies led to the now well-known Z-shaped 2D fingerprint.^42,43,66^ This highlights the role of structural dynamics or disorder of the structure, which is a key factor in interpreting the 2D-IR response of a protein. Disorder or dynamic motion contributes to the line widths of the bands from individual secondary structural elements. This means that, for example, two helical sections of similar length may give different spectra as a result of their dynamics. Thus, the flexibility of secondary structure elements contributes significantly to making the 2D-IR responses of proteins unique.^33^

In parallel with experiments, considerable effort was also being dedicated to developing computational models of protein 2D-IR spectra. These studies followed a similar pattern to the experimental approach in terms of learning to translate 2D amide I lineshapes into protein structures and *vice versa*.^38^ The details of the process have been discussed in detail elsewhere and are beyond the scope of this article but range from studies using Density Functional Theory to simulate spectra of small peptides and model structures^67–74^ to MD and quantum-classical approaches that were applied to larger molecules.^75–79^ The result has been the development of maps that enable the assignment of amide I mode frequencies based on the backbone geometry of the peptide links that are obtained from MD simulations.^37,80^ To these frequency maps are added coupling models that account for interactions between the amide groups arising from the folded structure of the peptide. Overall, the approach has been successful, and a series of usable models have been developed that can predict a 2D-IR spectrum from a protein structure with reasonable accuracy.^81^ Conversely, upon measuring a 2D-IR spectrum of a protein, the experimentalist can identify broad regions of the signature with specific elements of the structure.

In 2012 the first concerted attempt to derive the structure of proteins directly from a label-free 2D-IR spectrum was made by measuring the amide I 2D-IR spectrum of 16 proteins of differing secondary structure content.^41^ Singular Value Decomposition (SVD) analysis was used to quantify the amount of α-helix, β-sheet and unassigned elements of the structure. This approach tested the assumption that the protein amide I band is an additive combination of the contributions from different secondary structural elements. While this neglects interactions between secondary structure elements arising from tertiary and quaternary structures, the fact that couplings between residues dominate the shape and position of the band means that it is likely to be an accurate one. Indeed, when comparing the results with the structures of the proteins determined via crystallographic data the approach showed good agreement (accurate to ∼ 9%), which demonstrated proof of concept for such applications of 2D-IR.^41^ In particular, the accuracy of prediction achieved was found to be comparable to the gold standard for secondary structure analysis, Circular Dichroism spectroscopy (CD). The article also proposed that errors were in part due to differences between crystal structures, which are routinely measured, and the structure of the fully solvated, room temperature protein in solution, the latter being likely to be heterogeneous, as well as differing from the solid phase sample. A final source of potential error was cited as the lack of detailed understanding of the impacts of size on the spectra of secondary structure elements.

Continued development meant that this last question was subsequently addressed and a detailed understanding of the impact of a range of factors on the 2D-IR spectroscopy of secondary structure elements emerged. This included studies of the impact of α-helix length and β-sheet dimensions on the anharmonicity of the amide I band.^63,82^ A series of studies also investigated the impact of coupling on the transition dipole strength of a particular secondary structure element. This exploited the nonlinear nature of 2D-IR spectroscopy to show that coupling increases the 2D-IR signal size relative to the FT-IR response.^83,84^ This correlation between degree of coupling and the strength of the 2D-IR band has considerable implications for analytical studies as we shall see below.

Alongside the ability to determine absolute protein structure in a quantitative manner, the ability to quantify changes in structure caused by a specific event could also be a powerful measurement. For instance, one can conceive of experiments that wish to know the degree of structural change caused by ligand binding. In an effort to assess the suitability of 2D-IR for such applications a study used 2D-IR spectroscopy of the calmodulin protein as a function of temperature and calcium ion concentration to evaluate the degree of structural change.^85^ In addition to demonstrating quantitative agreement with CD methods, it was shown that 2D-IR could observe changes in α-helix content involving as few as 7–8 residues from a total of 148. This is perhaps an important observation as IR can be thought of as a relatively blunt instrument in comparison to other structure-based analytical tools, but this work provides evidence that the size and sensitivity of the amide I 2D-IR signature of full proteins lends itself to measuring relatively subtle changes in structure.

While the majority of studies applied computational methods as a means to analyze or extract additional insight from experiments, more recent approaches have begun to make the link between computational and experimental methods more synergistic. For example, Bayesian ensemble refinement has been applied to the tripeptide Ala–Ala–Ala in combination with FT-IR and 2D-IR spectroscopy.^86^ The aim was to derive structural insight into the conformational distribution of an intrinsically disordered peptide. This addresses an important class of protein that presents a significant challenge for structure-determination methods because of the presence of multiple, undefined but interchanging conformations. By using predicted spectra to guide the interpretation of experimental ones and determine how various secondary structures contribute to the spectrum of the disordered ensemble, this work shows the value of the combined technique for studying dynamic proteins. This example used isotope edited peptides but demonstrates the potential of the method. More recently, the advent of Machine Learning applications to 2D-IR data seeks to build on this progress and we return to these studies in a dedicated section below.

The ability to derive protein structural insight from 2D-IR spectra has led to a number of applications of label free-amide I IR spectroscopy. Examples include the use of amide I and II bands to study the structure of antifreeze glycoproteins.^87^ In this case the work focused on the contribution of the polyproline II (PPII) helix to the spectrum.

2D-IR spectroscopy of the amide I band has also been used to follow denaturation of protein structures. Addition of guanidinium ions was found to have a greater impact upon β-sheets than α-helices, based on the ability of the 2D-IR spectrum to clearly resolve the contributions of these structural elements to the signal (Figure 4).^88^

**Figure 4:**
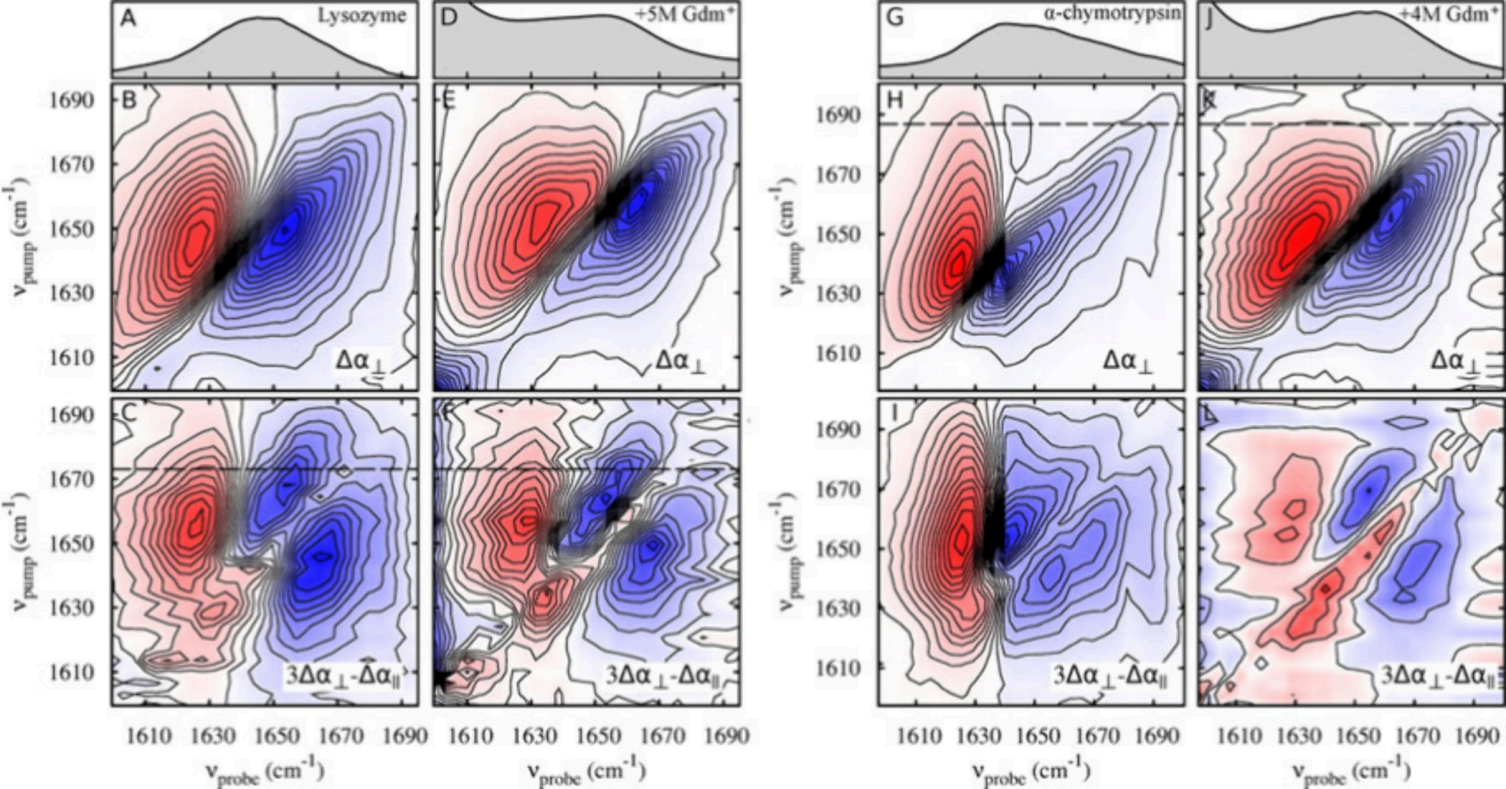
a) FTIR spectrum of lysozyme (solvent subtracted). (b) 2D-IR spectrum of lysozyme for perpendicular polarization of pump and probe pulses. Blue indicates a negative absorption change, and red indicates a positive absorption change. (c) Difference 2D-IR spectrum (3Δα_⊥_ – Δα_∥_) of lysozyme. (d) FTIR spectrum of lysozyme at a 5 M Gdm^+^ concentration. (e,f) Corresponding perpendicular polarization and difference 2D-IR spectra. (g) FTIR spectrum of α-chymotrypsin (solvent subtracted). (h) 2D-IR spectrum of α-chymotrypsin for perpendicular polarization of pump and probe pulses. (i) Difference 2D-IR spectrum of α-chymotrypsin. (j) FTIR spectrum of α-chymotrypsin at 4 M Gdm^+^ concentration. (k,l) Corresponding perpendicular polarization and difference 2D-IR spectra. The pump–probe delay of the 2D-IR spectra was 1.5 ps. The band at ∼ 1610 cm^–1^ in (d) and (j) is the high-frequency wing of Gdm^+^-^13^C^15^N_3_ absorption. Reproduced from ref 88. Copyright 2013 American Chemical Society.

An interesting development in terms of applications of the structural abilities of 2D-IR reported the use of antibodies tethered to a plasmonic surface which captured amyloid proteins from a flowing solution.^89^ This allowed 2D-IR interrogation of the structure of the fibrils that formed from the aggregation, or self-assembly, of the amyloid. This study, which will be discussed in more detail in a later section, neatly combines the specificity of antibodies for sample selection with the ability to increase sensitivity arising from the use of tethering and so increased local concentration of the analyte.^90^ Crucially, this approach also adds structural insight into immunological assays.^89^

Although we focus primarily on amide I 2D-IR spectroscopy, another related technique has shown considerable promise for the type of analytical applications that we consider here. Known as DOVE (doubly vibrationally enhanced) four-wave mixing or EVV (electron-vibration-vibration) 2D-IR spectroscopy, this approach generates a third-order nonlinear signal from two independently tunable IR pulses and a visible, readout, pulse.^91,92^ EVV 2DIR is thus a hybrid Raman-IR method but also generates spectra that are a map of coupled vibrational modes. EVV 2D-IR has shown potential for protein identification by identifying specific vibrational signatures in the spectra of amino acids such as tyrosine and phenylalanine.^93^ These identifying peaks were then compared to the spectra of 10 known proteins, showing that the characteristic peaks could bequantified using less than a minute of signal acquisition. It was thus argued that this approach could provide a viable route to optical proteomic analysis. Of further interest to protein analysis applications, it was shown that post-translational modifications in peptides can be detected by EVV 2DIR spectroscopy.^91^ These have not been tackled using all-IR 2D-IR methods and so offer an important complementary insight.

### 3.3 Biomolecular Interactions

Many biological processes depend on the formation of intermolecular contacts, whether through ligand binding or the self-assembly of clusters between diverse biomolecules. These processes can be transient and rely on dynamic changes in structure while the role of solvation can also be important. As such, the ability to observe and characterize these interactions in solution at biologically relevant temperatures would provide vital insights into the driving forces and functions of intermolecular interactions.

2D-IR spectroscopy has been widely used to study biological intermolecular interactions and this may prove to be one of the more fruitful avenues for analytical applications in the future given the combination of time resolution, structural insight and speed of data acquisition. Perhaps the largest body of work in this area has focused on the use of 2D-IR to study the formation of amyloid fibrils since 2008.^84,94–109^ In this article we do not seek to relate the molecular story that has been uncovered, rather we focus on the analytical method development that has arisen in parallel with these studies, the legacy of which is a suite of powerful techniques that can be used by the analytical 2D-IR spectroscopist.

The first of these advances introduced the use of automated 2D-IR spectroscopy to observe formation of amyloid fibers from human islet amyloid polypeptide (IAPP).^94^ In these experiments an IR pulse shaper was used to collect 2D-IR spectra in <1 s using phase cycling to minimize the effects of scattered light from the heterogeneous sample. Using continuous acquisition of spectra the increase of β-sheet like signatures was observed as the fibers grew, similar to a traditional stopped flow spectroscopy experiment. Subsequently, it was shown that similar measurements in the presence of lipid vesicles led to the presence of an intermediate structure demonstrating that membrane-catalyzed processes could be followed alongside structural resolution of evolving mixtures.^95^ A significant body of work evolved from this point in which isotopic labeling was introduced to develop local, site-specific information. Instead, we continue to focus on label-free applications such as a multidisciplinary study in which 2D-IR spectroscopy was combined with fluorescence assays, light scattering, circular dichroism and transmission electron microscopy to study the behavior of a compound that showed inhibitory properties, arresting IAPP fibril formation by the trapping of intermediate structures.^96^ This work highlights how analytical 2D-IR can be used to offer part of the answer to a complex problem. Similar multidisciplinary approaches were employed to study plaque formation caused by apolipoprotein following oxidation, which relates to atherosclerosis and cardiovascular disease,^98^ and to investigate the structural changes in alpha-synuclein amyloids (involved in the pathogenesis of Parkinson’s) caused by changes in ionic strength,^107^ N-terminal acetylation,^108^ and C-terminal truncation.^106^

Such multidisciplinary studies also revealed a potential route through which 2D-IR spectroscopy can contribute to the design of therapeutics, via a study in which time-resolved 2D-IR, alongside other biophysical and biological measurements, identified a toxic species arising from IAPP amyloid formation. Understanding their structural and chemical nature was cited as essential information to guide drug design strategies to combat the toxic steps.^99^

The transition dipole approach to studying peptide and protein secondary structures reported in 2012, was applied to understand structure formation during fibril aggregation. This approach relies on the fact that coupled structures lead to changes in transition dipole strengths, related to mode delocalization, to which 2D-IR is sensitive, but FT-IR is not. In this way it was possible to use the ratio of FTIR and 2D-IR spectral intensities to uncover structural differences between rat amylin when bound to micelles, membranes and in solution.^83,84,110^

All of the previous studies were carried out in the solution phase but a transition to making 2D-IR measurements in tissues was reported in 2017 with studies of aggregation of Crystallin proteins in eye lenses.^100^ Once again, the spectroscopy was used to detect the presence of amyloid fibers, which contribute to or are present upon cataract formation. The study was extended to human lenses in 2019.^102^

The most recent advance of analytical applications of 2D-IR for measuring molecular interactions via fibril-forming systems involves the use of polarization to accentuate the off-diagonal peaks of the spectrum.^111,112^ Thus, far, the spectroscopy of the β-sheet like fibrils had been dominated by the strong on-diagonal signals and their coupling patterns. It was however shown that by setting the correct relative polarization directions for pump and probe pulses it is possible to suppress the diagonal peaks revealing a complex set of off-diagonal resonances. These were found to be specific to different polymorphs formed during fibril formation, shedding light on the nucleation and kinetics of the aggregation via the structural resolution of 2D-IR spectroscopy.^112^

The methods produced during this process of development have found applications to other systems, showing their value as analytical methods. Examples include studies of F-actin filaments and their response to molecular crowding^113^ and the use of transition dipole methods to reveal changes in the aggregation of the cyclic antibiotic peptide Gramicidin-S.^114^

In addition to aggregated structures, 2D-IR spectroscopy has been applied in label-free form to protein clusters and complexes. Examples include a body of work examining the role of allosteric phenomena in protein complexes.^115–117^ While these studies ultimately used modifications to the proteins in order to photochemically initiate changes in structure, which falls beyond the remit of a review on analytical applications, they do highlight the ability of 2D-IR to extract information on multicomponent complexes via the amide I band and also used cross comparisons between related proteins to understand differences in behavior. These strategies could be extended to analytical studies.

A similar series of papers have been dedicated to understanding the structure and binding of the insulin dimer.^118–126^ A protein hormone that has a well-known role in regulating blood sugar levels, insulin operates via a complex set of oligomeric structures, within which the formation and dissociation of the insulin dimer is a key step. 2D-IR spectroscopy has been used to investigate the relationship between the structure and amide I spectroscopy of the insulin dimer.^119^ Of particular interest was the observation that elements of the amide I band became delocalized over both monomer units arising from the formation of an intermolecular antiparallel β-sheet. This shows that the 2D-IR signature is sensitive to the formation of complexes and so can be applied to observing and measuring interprotein interactions. As mentioned in relation to earlier studies of protein systems, an important role was played in these studies by computational spectroscopy and spectral predictions, which helped to assign key features.^126^ These spectral signatures were also employed in time-resolved studies of insulin binding using temperature jump initiation,^121–125^ which lie beyond our current scope.

A clear opening for the structural insight of 2D-IR spectroscopy would seem to lie in the field of drug design. The ability to measure structure-related data quickly and in a label-free manner under room temperature, solution phase conditions could contribute valuable insight into drug candidate selection and optimization. Demonstrations of the potential of the technique have used 2D-IR spectroscopy of proteins related to tuberculosis (InhA) in complex with drug molecules,^127^ revealing changes in the 2D-IR spectrum upon binding. Even in cases such as this, where significant changes in protein structure do not accompany binding, the presence of the drug leads to a variation in the amide I profile.^127,128^ This was attributed to differences in dynamics of the protein–drug complex, which can influence vibrational couplings and so lineshapes and transition dipole moments. Thus, 2D-IR has proved to be a sensitive tool for detecting drug presence. Furthermore, the nature of the changes in the spectrum appear unique to the combination of protein and drug.^128^ Of particular interest, it was revealed that mutations of the InhA protein that are implicated in drug resistance in tuberculosis, a major global health problem, were found to have different spectral responses to drug binding and so implicated protein dynamics in the mechanism of resistance.^127^

In a further study, the structural dynamics of different isoforms of the cytokine protein IL-17,^129^ which is associated with autoimmune diseases, were found to vary in response to drug binding. The range of structural rigidities observed for IL-17 variants and their complexes also correlated with receptor binding ability, offering a route to informing drug design strategies through measurements of dynamics (Figure 5).^129^ Through cases such as InhA and IL-17 it has become clear that 2D-IR spectroscopy offers a route to measuring a structurally and dynamically sensitive fingerprint that varies in response to drug binding. Thus, one can envisage screening campaigns using different drug candidates against a target protein, searching for molecules that induce a specific change in the fingerprint. Such an approach would be faster and use less material than an NMR campaign and would not need isotopic enrichment or substitution. Furthermore, neither crystal formation nor other complex sample preparation is required. The 2D-IR screening approach has been demonstrated using small molecule binding to DNA sequences in a study that piloted the use of principal component analysis based tools for differentiating and grouping spectra.^130^ One envisages that such approaches will increasingly be required to keep pace with the speed of data collection.

**Figure 5:**
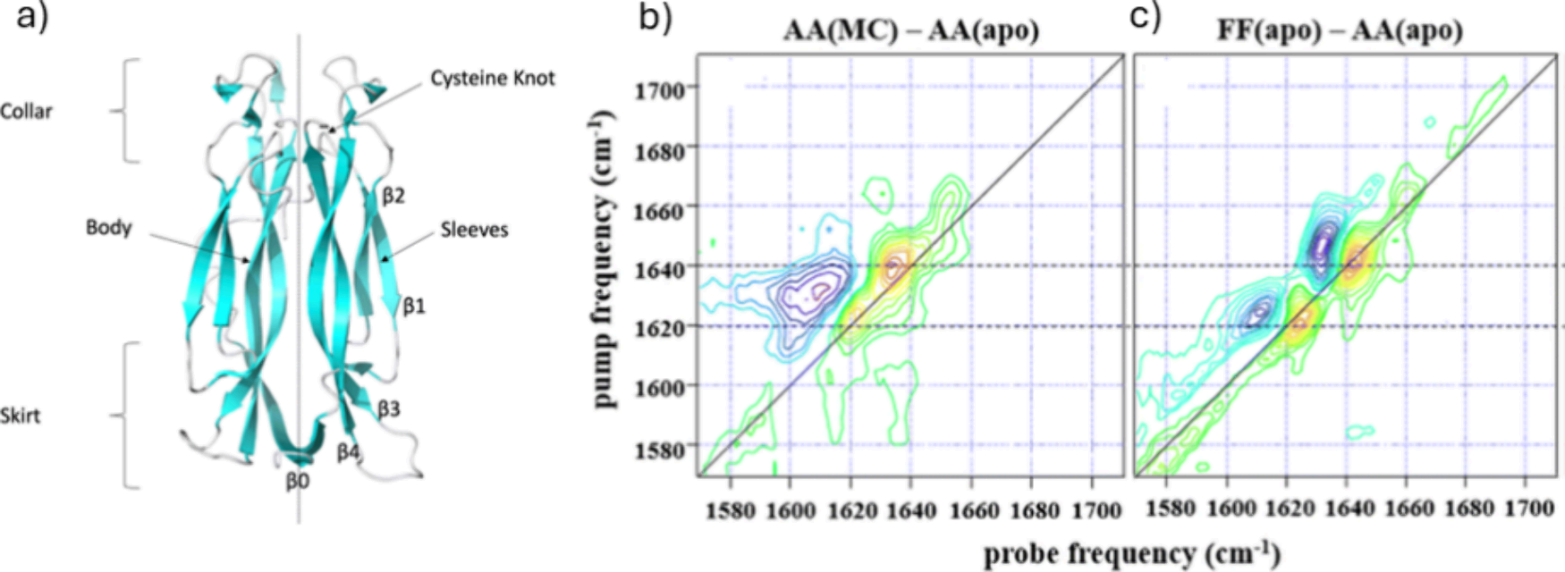
a) Structure of IL-17 homodimer. b) and c) show difference 2D-IR spectra between b) IL-17AA with a macrocyclic drug (MC) bound and the apo form and c) between the FF and AA forms of IL-17. The similar patterns reveal that drug binding to IL-17AA modulates the dynamics of the protein to mimic the more rigid FF form of the protein. Reproduced from ref 129. Available under a CC-BY-NC-3.0 license. Copyright Neil T. Hunt, Frederick W. Muskett.

EVV 2DIR methods have also been applied to detect drug binding to proteins. Specifically, a subset of peaks were uniquely assignable to protein–drug interactions between the FGFR1 kinase and a drug designed to target it.^131^ The different information content of EVV 2DIR, which does not utilize the amide I band was able to shed light on the structure of the bound drug molecule. By finding that none of the peaks assignable to couplings of vibrational modes of the drug molecule changed substantially upon binding, it was inferred that the structure of the bound drug is virtually identical to the free molecule.

Finally, 2D-IR spectroscopy has also been used to understand the interaction between ions and proteins. In a similar manner to potential applications to drug binding, such studies seek to understand and produce spectral signatures for specificity in ion binding.^85,132–136^ This knowledge would be expected to inform development of bioinspired tools for rare ion recovery in recycling applications for example.

### 3.4 The Role of Water and Physiological Sample Analysis

The preceding studies, and indeed the majority of 2D-IR investigations of the amide I band of proteins have been carried out in deuterated water (D_2_O). This reflects the problem posed by the H–O–H bending vibrational mode of H_2_O, which occurs at 1650 cm^–1^ and so coincides directly with the amide I band. Deuteration removes the resonance by shifting the bending vibrational mode down in frequency to around 1250 cm^–1^, away from the amide I region. While a practical solution that has enabled significant progress in our understanding of the amide I band and proteins more generally, it is inescapable that this isotopic labeling also has an impact upon the accuracy of the measurement.^137^ The kinetic isotope effect in proteins tells us that switching to deuteration affects the functioning of proteins via their reaction rates and dynamics. D_2_O ‘H-bonds’ differ in strength to those in H_2_O, which may shift the delicate balance of forces that underpin the structure, dynamics and interactions of a protein. Combinations of these effects can be seen through the change in the amide I frequency by around 10 cm^–1^ upon deuteration.^138^ Finally, removing the energetic resonance between solvent and protein may have implications for vibrational energy redistribution, such that the molecule that we measure in D_2_O is just a first approximation, albeit a good one, of the one present under physiological conditions.

As mentioned above, 2D-IR spectroscopy provides a route to circumvent this problem because the amide I band has a molar extinction coefficient some 2 orders of magnitude larger than that of the water bending mode.^25,139^ This means that, provided the sample is sufficiently optically thin as to allow passage of the IR pulses through the sample (∼ 3 μm), 2D-IR accentuates the protein response over that of the water. Furthermore, as the amide I vibrational lifetime is some four times longer than that of the water bending mode (800 fs vs 200 fs)^140–142^ the water signal decays to leave the protein signal behind. Finally, H_2_O gives rise to a small thermal signature that appears on ps time scales after both the protein amide I and water bending modes have relaxed. This thermal signature has been shown to scale linearly with the 2D-IR protein signature, allowing it to be used as a normalizing factor for comparing between different samples by compensating for experimental factors such as laser power, quality of beam overlap and sample concentration and path length.^143^

This approach has led to a range of studies of proteins in H_2_O, primarily focusing on the measurement of proteins in blood serum samples.^25,128,143–148^ These applications will be covered in more detail in a section on biomedical applications below, but 2D-IR spectroscopy has been used to measure protein concentrations,^25^ protein–small molecule interactions^25,128,147^ and to differentiate clinical samples based on their protein content, using the amide I band.^148^

The ability to measure protein 2D-IR spectra in water has also extended beyond blood serum measurements. For example, insulin fibril formation has been compared in D_2_O and H_2_O.^105^ It was observed that fibril formation is significantly slower in deuterated solvents with the presence of differing nucleation structures noted. In a similar study on collagen the reverse situation was observed, with collagen assembly occurring faster in D_2_O than H_2_O.^149^ This was attributed to the D_2_O sample being less hydrated leading to a greater number of nucleation centers. The structural aspects of the study were based upon circular dichroism, IR absorption and 2D-IR, however, while 2D-IR studies were carried out in D_2_O the equivalent ones in H_2_O were not reported.

## 4 Complex Matrices

### 4.1 Mixture Analysis

It was described above that the 2D-IR fingerprint generated by a particular molecule offers an opportunity to identify its presence within a complex mixture, either to measure its concentration or gauge the nature of the interactions with other components. Such an application was first demonstrated in 2003 but has found more applications in recent years.^32^

An example that extends the theme of biological molecules from the previous section used 2D-IR spectroscopy to examine therapeutics that are made from cocrystallizations of two drug molecules.^150–152^ In the example studied it was reported that sacubitril allisartan calcium, which is used to treat hypertension and heart failure, is known to exist in two crystalline forms with the same molecular composition, but with only one of the structures known.^152^ 2D-IR experiments targeting carboxylate and amide groups investigated the structures of the Ca^2+^ coordination complex of the crystal unit cell. The results highlighted a structural variation between the crystalline forms arising from the ratio of bidentate to bridging carboxylate groups. Furthermore, differences in spectral diffusion dynamics and energy transfer rates were used to infer differences in local structural rigidity and intermolecular distances. It was stated that this information aids in understanding of the stability and release mechanisms of the drug molecules in cocrystalline formulations. In addition, as with the idea of drug candidate screening introduced above, one can imagine the use of high throughput 2D-IR spectroscopy for quality control, whereby the 2D peak pattern could be used to identify which crystalline configuration is present during preparation. In terms of sample preparation, the authors used liquid suspensions of the crystals measured in the traditional 2D-IR geometry.

In addition to biology or biochemistry, the structure and interactions of proteins have considerable significance in the food industry where they play a role in both taste and texture of foodstuffs. To address this question 2D-IR spectroscopy was applied to study milk proteins in the presence of caffeine, both in isolation and in coffee.^153^ The underlying question is of relevance because milk proteins theoretically interact with many organic molecules in tea and coffee, with the possibly that these interactions may affect the availability or efficacy of bioactive molecules such as polyphenols, which have been linked to antioxidant properties, or caffeine. The authors used 2D-IR spectroscopy to separate spectral contributions from the amide I band of milk proteins and those from caffeine, which overlap strongly in 1D spectroscopy. Indeed, the paper also highlighted the elevated sensitivity of 2D-IR as compared to FT-IR through the ability to detect peaks due to vibrational modes of caffeine that were barely visible in absorption spectroscopy.^153^ The study also highlights potential difficulties in applying 2D-IR to complex systems, however. Water is a problem for studies of natural milk and coffee while scattering from the milk emulsion (fats and proteins in water) could be problematic. Scattering could be avoided by the use of phase cycling,^94^ but the 2D-IR spectroscopy was performed using samples of milk and coffee that had been diluted 10 times with D_2_O. As well as departing from the ‘physiological’ situation, this could also lead to complex behavior of the proteins present, for example through preferential or partial solvation. Interestingly, the study showed no interactions between caffeine and milk proteins either in terms of direct interactions or through the influence on spectral diffusion dynamics of the amide I band.^153^ In respect of the latter observation, the authors also discussed the difficulty of applying spectral diffusion to complex mixtures where peak overlap influences spectrum more than line shape change. Despite the seemingly negative result, the article represents a first step in applying 2D-IR to food-related problems and shows the potential of the method. Future studies to unravel the contributions to the amide I response of the multiple proteins that are present in milk would be an interesting development.

Another application of 2D-IR spectroscopy under conditions which entail a mixture of species studied the secondary structure of peptides implicated in biomineralization.^154^ This process whereby organisms produce organic–inorganic composite materials to form structures including exoskeletons, shells and bones is attractive as a template for biomimetic applications because it takes place quickly, at room temperature under mild conditions of neutral pH and atmospheric pressure. Potential areas of application stretch from medicine to microelectronics. In the study of R5, a 19-residue peptide originating from diatoms, where it is involved in the transformation of silicic acid into biosilica nanoparticles, 2D-IR spectroscopy was used to follow the process of biosilicification. By comparing 2D-IR spectra of the peptide in D_2_O in the presence and absence of silica with spectral predictions based on MD results it was possible to shed light on the structures formed by the peptide. As a short peptide, R5 is somewhat reminiscent of an intrinsically disordered system, so MD structures were grouped into clusters and linear combinations of the predicted 2D-IR spectra for each cluster were used to infer the origins of the spectral responses recorded. While there is likely to be an element of uncertainty in the outcome, it was possible to conclude that unbound R5 favors β-strand structures among others referred to as atypical. Silicification led to a decrease in β-strand structures alongside an increase in turn and bend configurations. The latter was assigned to the presence of silicate within the H-bond network of the peptide.^154^

Cells present a significant challenge to 2D-IR studies, not only because they represent a complex mixture of biological molecules, making chemical resolution difficult, but also because they are generally of a size that leads to severe scattering artifacts during data collection. The first instance of 2D-IR spectroscopy being applied to cells was reported in 2004, albeit in the form of the closely related spectrally resolved stimulated vibrational echo measurements.^155,156^ In this case the dephasing dynamics of the CO stretching mode of hemoglobin–CO was measured inside human red blood cells. Perhaps surprisingly, it was shown that the dynamics of the protein as sensed by the CO ligand are the same when inside the cells as are found in aqueous solution, despite differences in the absorption spectra showing some impact of the cell on stretching frequency. Later it was shown that the dynamics sensed inside the cell were also independent of viscosity.^156^

A more analytical application of 2D-IR in cells exploited the 2D amide I fingerprint for the detection and classification of bacterial spores.^23^ Comparing spectra of *Bacillus atrophaeus* and *Bacillus thuringiensis* spores as dry films on CaF_2_ windows showed the presence of diagonal and off-diagonal peaks arising from the sporulation biomarker calcium dipicolinate. Furthermore, differences in the amide I region allowed differentiation of the two types of spore. With a view to future applications, the ability to detect the spores on surfaces, in suspensions as well as in dry films was demonstrated. In these two examples of experiments on cells, phase cycling was used to reduce the effects of scattered light. In the first instance a mechanical approach was employed^155,156^ while the latter benefitted from the advances brought by pulse shaping technologies.^23^

Moving on briefly from biological systems, 2D-IR spectroscopy has also found analytical applications in examining energetic materials, or explosives. In addition to opening up a different avenue of enquiry, this also introduces us to alternative sample presentations such as thin films.

In an example from 2017, 2D-IR measurements of Pentaerythritol tetranitrate (PETN) films exploited the ability to measure vibrational energy transfer and relaxation mechanisms.^157^ PETN itself is a common secondary explosive that has been used to provide information relating to shock initiation and energy propagation in energetic materials. The experiments showed an ultrafast reorientation of transition dipoles in PETN films that were assigned to intermolecular energy transfer. In addition, an energy transfer pathway with a 2 ps time scale was revealed via 2D-IR off-diagonal peaks. Supporting the experiments with calculations suggested that intermolecular couplings in the crystal approached or exceeded intramolecular couplings and that energy transfer was taking place between states that involve multiple PETN molecules. As different structures and packing in crystals can lead to different impact sensitivities, this study allowed the 2D fingerprint to be used to identify and differentiate signatures that may relate to the macroscopic behavior of the materials.

In a later study, the structural and vibrational-energy transfer dynamics of cyclic nitramine hexahydro-1,3,5-trinitro-s-triazine (HHTT) were investigated with 2D-IR.^158^ In this case the samples studied comprised a solution of HHTT in DMSO and microcrystals dispersed in paraffin. Both intra- and intermolecular vibrational energy transfer routes were reported in the microcrystal which was disrupted in solution, showing the importance of the crystal structure in determining energy relaxation pathways. Similar to PETN, intermolecular electrostatic interactions and H-bonds were associated with intermolecular effects. More recently a theoretical study has simulated 2D-IR spectra for a range of different energetic materials, which should form a basis for future experiments.^150^

### 4.2 Biomedical Applications

Biomedical samples offer a special class within the complex mixtures category. This arises partly because of the multimolecular nature of the samples but also the range of different materials that may constitute a biomedical sample. Examples referred to below range from solid tissue, to biofluids via ocular lens materials, each of which provide specific challenges. The added dimension of difficulty when dealing with biomedical samples is that the ultimate aim will be to extract clinically meaningful data from the spectroscopy in a manner that may be useful to patients or clinicians.

The first applications of 2D-IR spectroscopy to samples of direct biomedical relevance studied the amide I band of blood serum.^25^ Blood serum, blood with the red blood cells and clotting factors removed, is essentially a complex biomolecular solution containing a large number (hundreds) of proteins spanning a wide range of concentrations (pM-mM) alongside phospholipids (fats), sugars, nucleic acids and minerals, alongside other species. As serum comes into contact with most of the major organs it offers an unparalleled molecular window on metabolism and so the health of the body. This molecular complexity also means that serum presents a particularly difficult analysis problem. While techniques for measuring individual protein concentrations accurately and quickly exist, the ability to measure a broad snapshot of protein levels does not. Conversely, although single molecular biomarkers can be useful for diagnostic studies, the presence of disease leads to many changes in the molecular profile of serum and it is conceivable that other key biomarkers could be identified which involve concerted changes in groups of proteins or across more than one molecular species, for example fats and proteins.

It has been shown that 2D-IR spectroscopy offers a route to such information, building on the ability to measure proteins in aqueous fluids, it was shown that 2D-IR could be used to measure changes in concentration of some of the most abundant proteins via studies in which proteins were spiked into serum samples.^144,146^ In these experiments 2D-IR measured the amide I response of the serum sample, which encompasses the amide I band of all constituent proteins. Although IR spectroscopy is not capable of particularly high sensitivity, in comparison to methods such as Raman or fluorescence, it has been established that amide I spectroscopy of serum could be used to provide data on fluctuations in the concentration of the 10–15 most abundant serum proteins, which would be a considerable advance on current methods.^159,160^ Data analysis represents a barrier to this technology because the contributions from individual proteins overlap, but recent applications of machine learning methods using both experimental and simulated 2D-IR data (which we discuss in more detail below) suggest that this combination could be a powerful future direction. Such applications learn to either recognize/quantify the contributions of individual components of the mixture or categorize the sample into a group according to the spectrum.^148,161–164^ Applications to serum samples are in their infancy^148^ but, as data science approaches and 2D-IR data libraries continue to improve and evolve, there is considerable potential for a 2D-IR blood test.

In addition to protein levels and patient sampling, 2D-IR spectroscopy in serum has been used to measure drug binding to serum proteins.^128^ In this case, the presence and levels of four different drugs were measured via their binding to serum albumin. This study was the first use of machine learning methods for pattern recognition in 2D-IR spectroscopy, which was able to identify the presence of drugs by their impact on the serum albumin component of the 2D-IR spectrum. This work also revealed the potential sensitivity gains involved in using the amide I signature of serum albumin for such a study. The changes in the serum 2D-IR spectrum upon adding drug molecules were measurable at physiological drug concentrations (few μM) far below what would have been detectable if the drug were to be detected directly. The strength of the protein signal thus served to enhance the sensitivity of the measurement. Such measurements could have relevance for studies ranging from understanding drug behavior in the bloodstream to unravelling the complex binding of serum albumin and measuring drug metabolism and pharmacokinetic (DMPK) profiles.^128^

Perhaps the most directly biomedically relevant 2D-IR study using serum has measured the amide I band of patient serum samples.^148^ It was shown that machine learning in combination with 2D-IR spectroscopy was able to classify blood serum samples collected from 40 patients who had received treatment for melanoma according to the presence, absence or later development of metastatic disease. This is achieved by the algorithm learning key spectral markers for each clinical group from a training set of patient samples, before using this to categorize new patient spectra into one of the groups. Evaluating the accuracy of the results via the area under the receiver operating characteristic curve (AUROC, where a value of 1 indicates ideal sensitivity and specificity) produced values of 0.75 and 0.86, showing the ability to identify samples from patients who were radiologically cancer free and with metastatic disease, respectively. Perhaps most excitingly, the model was also able to classify samples from a third group of patients (AUROC = 0.80) who were radiologically cancer-free at the point of testing but would go on to develop metastatic disease within five years. This ability to identify post-treatment patients at higher risk of relapse from a spectroscopic measurement of biofluid protein content showed the potential for hybrid 2D-IR-machine learning based risk stratification methods for melanoma patients.

The use of 2D-IR spectroscopy to study the structure and formation of amyloid fibrils was discussed above and that work has obvious links to biomedical studies. Of particular relevance to this section are studies that have extended measurements of fibril formation into blood serum.^103^ In this case it was necessary to deplete the serum of the major protein constituents serum albumin and the immunoglobulins before replacing H_2_O with D_2_O. Different fibril polymorphs were observed, which were attributed to the presence of the remaining serum biomolecules.

In addition to liquid phase samples, 2D-IR spectroscopy has been extended to biomedically relevant imaging studies. The approach sought to build on methods like hyperspectral FT-IR imaging, in which an IR spectrum is recovered at each point in a sample, to enable the 2D-IR response of a sample to be studied. The method was applied to study tissue samples in the form of formalin-fixed mouse pancreatic tissue.^18^ By scanning the sample relative to the two laser beams (pump and probe) that are used to measure 2D-IR spectra, a hyperspectral image was produced at points separated by 50 μm over a region measuring between 1 and 5 mm in a given direction. Typically, the images consisted of more than 950 spectra and provided a spatial resolution of 100 μm^2^. The samples were formalin-fixed and paraffin embedded sections through a mouse pancreas. This standard process in bioimaging cross-links proteins to preserve their structure. 2D-IR images were obtained at time points of three, eight and 48 weeks from the point of collection. Using the amide I band the authors created maps of 2D-IR spectral parameters related to protein structure, including diagonal peak ratios, the amplitude of off-diagonal peaks and anharmonicities. This reduced the data set to interpretable hyperspectral images and histograms allowing comparison of the information from the samples. It was concluded that the extent of β-sheet formation increased with time, suggesting that formalin fixation did not fully arrest the protein structural changes in the pancreatic tissue, an effect that was not observed in lenses.^18^

Overall, this extension of the power of 2D-IR to tissue samples shows the potential for amide I spectroscopy to be applied to understand protein content and structure under a range of physiologically relevant conditions and in a variety of biomedical samples.

The use of EVV 2DIR spectroscopy for tissue imaging has also been explored. In this case, preliminary results were presented showing a mouse kidney section that had been stained with hematoxylin.^91^ In the EVV application specific cross peaks arising from protein CH_3_ vibrational modes and from hematoxylin were used to obtain two different image visualizations by spatially mapping the peaks as a function of position within the sample. As hematoxylin stains mainly the cell nuclei and ribonucleic acids the two images provided different pieces of information.

## 5 Analytical Method Development

### 5.1 2D-IR Diffusion-Ordered Spectroscopy

The 2D-IR spectra of mixed samples are often difficult to analyze due to spectral overlap of the component 2D-IR spectra. One way of separating the total 2D-IR spectrum into its component spectra is by recording a series of 2D-IR spectra at different waiting times, and using differences in the component *T*_1_ lifetimes to extract the component 2D-IR spectra (obtained as decay-associated 2D-IR spectra from a global least-squares fit),^165^ but this only works if the component 2D-IR spectra do not depend on the waiting time, which is generally not the case. An efficient and more generally applicable way of addressing the spectral-overlap problem is 2D-IR diffusion-ordered spectroscopy (2DIR-DOSY), in which the 2D-IR spectrum is stratified based on the diffusion coefficients of the components present in the solution. The diffusion coefficient is inversely proportional to the hydrodynamic radius of a molecule or particle (by the Stokes–Einstein equation) so, provided that the components have different sizes, in a 2DIR-DOSY spectrum the total 2D-IR spectrum is cleanly separated into the component spectra, sorted by size (Figure 6). This experimental method is inspired by NMR-DOSY,^166^ but its experimental realization is different: the sample solution and pure solvent (or buffer solution) are injected parallel into an IR-transparent channel, and the subsequent diffusion of the solutes into the initially solvent-filled half of the channel is tracked by positioning the IR focus in this solvent-filled part and measuring a time-series of 2D-IR spectra (Figure 6a). The component spectra appear one by one in a cumulative fashion (Figure 6b), with a time-dependence determined by their diffusion coefficient. The time-dependent 2D-IR spectrum can be converted into a diffusion-coefficient-dependent 2D-IR spectrum,^167^ resulting in a 3D-IR spectrum with diffusion coefficient (or equivalently, size) along the third axis, and in which species with different sizes appear at different positions along the third axis (Figure 6c). This method has been used to investigate mixtures of amyloids and monomeric proteins successfully.^168^

**Figure 6:**
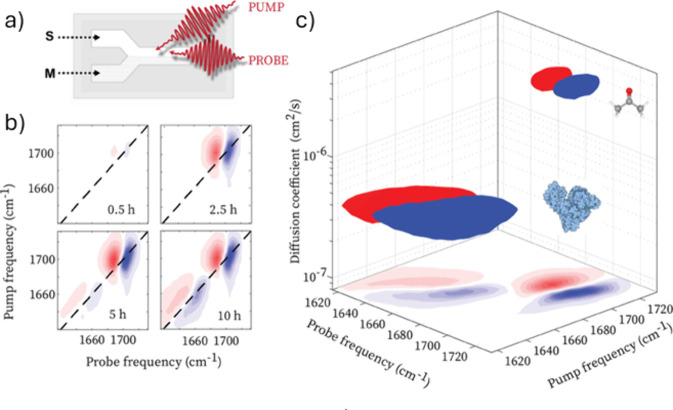
2D-IR Diffusion-Ordered SpectroscopY (DOSY) of a mixed solution of bovine serum albumin and acetone in D_2_O. (a) the sample solution and the solvent are injected in parallel into an IR-transparent channel (laminar flow). After injection, the solute molecules diffuse into the solvent-filled part of the channel, at the edge of which the 2D-IR focus is positioned; (b) As the different solutes diffuse into the 2D-IR focus, the 2D-IR spectrum changes accordingly; (c) The time-dependent 2D-IR response can be converted into a 3D-spectrum, with the diffusion coefficient (or equivalently, size) along the third axis. Species with different sizes appear at different heights in this 3D spectrum. Reproduced from ref 167. Available under a CC-BY-4.0 license. Copyright Giulia Giubertoni, Sander Woutersen.

### 5.2 Improving the Sensitivity of 2D-IR Spectroscopy

The above overview shows that 2D-IR spectroscopy can provide information on biomolecular structure in solution that may be difficult to obtain with other methods. Compared to protein NMR, the structural information content is less, but it can be obtained at lower concentrations, for proteins of any size, and with short measurement times. However, for analytical standards, the concentrations required for recording a 2D-IR spectrum are currently still very high. The lowest concentration of amide units at which a reliable 2D-IR spectrum can be measured is currently around 10 μM, which corresponds to a protein/peptide concentration of about 1 g/Liter (assuming an average amino-acid molar weight of 100 g/mol), e.g. 2D-IR spectra of 0.8 g/Liter solutions of glycine can be measured without problems.^11^ However, the concentrations of peptides that are measured in, for instance, HPLC-MS are many orders of magnitude lower than this and, at the moment, it would seem that hyphenating HPLC with 2D-IR spectroscopy will not become standard practice any time soon. In this section, we therefore consider approaches that are being explored to improve the sensitivity of 2D-IR and so allow for lower sample concentrations to be measured. As the issue lies in maximizing the experimental signal-to-noise ratio, the two main approaches being explored are signal enhancement to boost the measured response and noise reduction to improve sensitivity. We will discuss recent developments in each of these directions.

### 5.3 Surface Enhancement

Similar to Surface-Enhanced Raman Scattering (SERS),^169^ infrared absorption can be strongly enhanced when molecules are adsorbed onto noble-metal layers (Surface-Enhanced InfraRed Absorption or SEIRA). For linear infrared absorption, typical enhancement factors are on the order of 10 to 100.^170^ As explained by Osawa,^170–172^ this amplification of the absorption involves two mechanisms: electromagnetic and chemical. The former mechanism involves amplification of the near field around small (nanometer size) metal islands or roughnesses on the surface (the amplification strength depending on the morphology of the surface).^172^ The latter involves an increase in vibrational transition-dipole moment due to electronic interaction of the absorbed molecules with the metal surface, an effect that depends strongly on the chemical structure of the adsorbed molecules. Importantly, the near-field amplification effect decays rapidly with increasing distance from the surface, and mainly occurs at distances of up to a few nm from the surface.^170^

The 2D-IR response depends quadratically on both the electromagnetic field intensity and the vibrational absorption cross section (the linear response is proportional to (**μ**_**01**_ • ***E***)^2^), the 2D-IR response to (**μ**_**01**_ • ***E***)^4^), so the surface-induced signal amplification should be much larger for 2D-IR than for linear IR spectroscopy, and this enhancement has been extensively investigated.^173–177^ To quantify this enhancement, Hamm et al. defined an enhancement factor as (*S* ^2DIR^_surf_/*A*_surf_)/(*S* ^2DIR^_bulk_/*A*_bulk_), where *S* ^2DIR^ is the 2D-IR-signal intensity and *A* the linear absorption, in bulk and at the surface. This way of quantifying the enhancement has the advantage that it is independent of the surface coverage, which is often difficult to determine.^174^ In an early study, Donaldson et al. observed an enhancement factor of about 100 in the 2D-IR response of suspensions of amide- and carboxylate-capped aggregated gold nanoparticles.^173^ Later experiments used attenuated total reflection (ATR) 2D-IR, in which the pump and probe pulses travel through an ATR crystal and hit the gold-covered surface at an angle above the Brewster angle, so that the evanescent waves of the pump and probe pulses interact with the medium above the ATR crystal. Covering the ATR crystal with a gold layer of 1 nm average thickness can give rise to enhancement factors of up to ∼ 50 (Figure 7) for a monolayer of *para*-mercaptobenzonitrile adsorbed onto the gold (for other, nonaromatic adsorbed organic compounds the enhancement was less).^174^ Experiments using a transmission or reflection configuration (incident beam angles slightly below the Brewster angle) resulted in similar enhancement factors.^177^ These measurements were performed on an 18-residue cysteine-terminated peptide tethered to a gold surface and (in contrast to the most other studies) fully hydrated by a water layer. For thicker gold layers (3 nm on average), percolation of the gold occurs so that surface plasmons can be excited, leading to enhancement factors of more than 450.^175^

**Figure 7:**
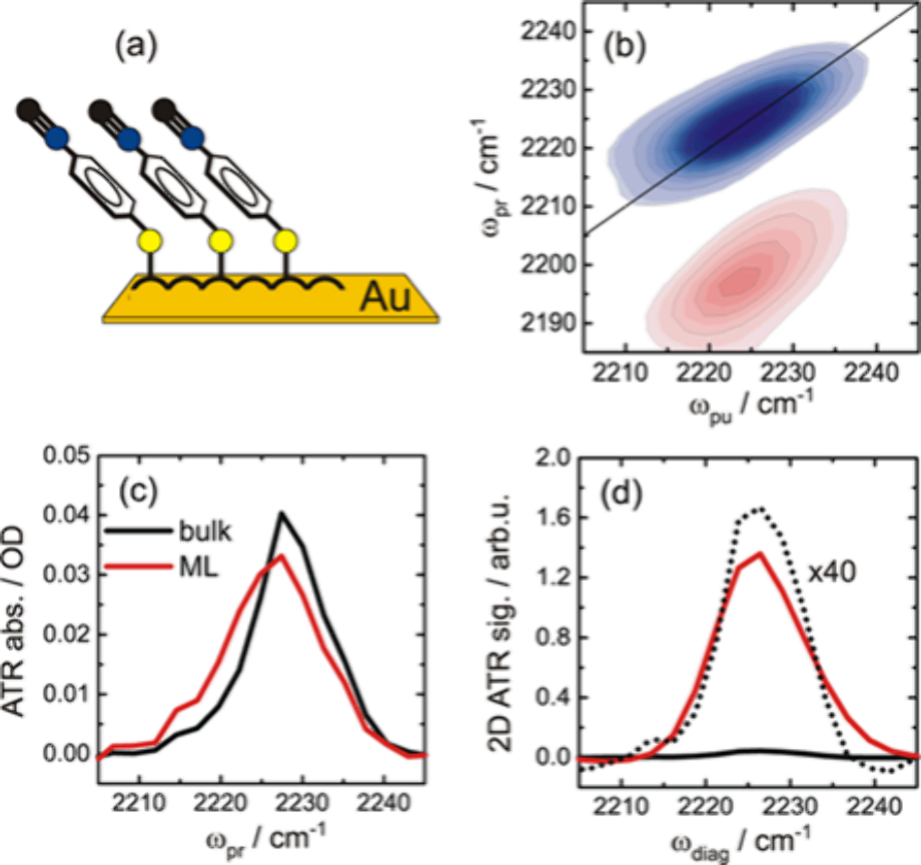
2D-IR signal enhancement by a thin (1 nm average thickness) gold layer. (a) The sample is a para-mercaptobenzonitrile monolayer covalently attached to the gold layer; (b) 2D-IR-ATR spectrum (CN-stretching mode) for a waiting time of 5 ps; (c) ATR-IR spectrum of the monolayer (red curve) compared to the ATR-IR spectrum of a 0.1 M solution; (d) diagonal cuts of the 2D-IR ATR spectra of the same two samples (same coloring of the curves), showing a 50-fold enhancement factor of the 2D-IR signal. Adapted from ref 174. Copyright 2016 American Chemical Society.

In addition to metallic surfaces, the use of deliberately nanopatterned surfaces to increase sensitivity has also been reported. This is driven by the development over recent decades of lithography methods able to produce well-defined nanoscopic structures that now make it possible to construct arrays of nanostructures that are tailored to enhance IR and 2D-IR signals. In particular, it is possible to construct arrays of gold nanoantennas with plasmonic resonance frequencies (determined by their size and shape) that match those of the molecular vibrations under study, making very large signal amplifications possible for both linear^178–181^ and nonlinear IR^181–184^ spectroscopy by relying on plasmonic resonances rather than surface roughness to enhance the local electromagnetic field. The linear absorption signal of protein monolayers deposited on such structures has been reported to increase by up to 10^5^,^179^ and the third-order nonlinear signal of W­(CO)_6_ in a PMMA film by up to 10^7^.^181^ Instead of plasmonic resonances, one can also use dielectric periodically structured metasurfaces with the appropriate resonance frequencies to amplify linear IR signals. In this way, using a Si layer with a periodic structure of holes deposited on CaF_2_, 1-to-2 order-of-magnitude enhancements of the linear IR response of a dilute solution of W­(CO)_6_ have been achieved.^185^ An important advantage of this latter approach is that the amplification effect extends to much larger distances (∼0.5 μm) away from the surface into the solution,^185^ which makes it possible to measure on dilute solutions rather than deposited/tethered layers. This seems promising as a way to amplify 2D-IR signals of dilute solutions, but unfortunately two-photon absorption by the Si makes 2D-IR spectroscopy difficult for the antenna array used in ref 185.

Surface-enhanced 2D-IR using plasmonic resonances seems a promising way to increase the sensitivity of 2D-IR spectroscopy, but a drawback for the analysis of solutions is the limited spatial extent of the amplification. Neubrech et al. showed that for plasmonic gold nanoantennas, the amplification for linear IR becomes negligible beyond 100 nm.^186^ Of course in cases where the biochemical analysis involves attaching solute molecules to the surface, the surface-specificity is an advantage. For surface-enhanced linear IR spectroscopy, several types of such bioanalytical applications have been reported (for a concise early overview, see Ataka and Heberle).^187^

Recently, plasmonic signal enhancement was extended through construction of a 2D-IR immunosensor. We briefly discussed the use of antibodies as a route to analyzing protein structure above. This application takes inspiration from immunoassays where the presence and concentration of an antigen (protein, hormone, drug) in a sample solution is detected by its binding to a specific antibody. By attaching the antibodies to a gold surface or to a surface covered with plasmonic antennas, it becomes possible to detect the binding of the antigens to the antibodies using surface-enhanced infrared spectroscopy. Using linear ATR-IR spectroscopy, Adato and Altug could observe and distinguish the high, weak, and zero-specific binding of different proteins to biotin-labeled antirabbit antibodies attached to a plasmonic nanoantenna array located inside a flow channel, and they could even observe water displacement during binding events.^180^ Recently, the first surface-enhanced 2D-IR-immuno-sensor was reported (Figure 8).^89,177^ Covering a plasmonic gold surface with polyclonal antibodies targeting human islet amyloid polypeptide (IAPP), it was possible to capture fibrillar IAPP present in a solution flowed over the immunosensor and measure its 2D-IR spectrum. Since the antibodies were much larger than IAPP it was necessary to subtract the 2D-IR-background signal of the former (1–3 mOD in magnitude) from the total spectrum to obtain the IAPP spectrum (10–100 μOD in magnitude), a procedure for which the use of a flow cell is advantageous since it minimizes systematic subtraction errors that might otherwise occur when exchanging samples.^89^ Interestingly, the 2D-IR spectrum of the antibody-bound fibrils (Figure 8e) differs from those in bulk solution (Figure 8f), a difference which the authors attribute to a preference of the antibody for a subset of aggregate structures. Using isotope-labeled IAPP, they could even show that the antibody binds two different polymorphs. This type of structure-sensitive immunoassay using plasmonically enhanced 2D-IR should be widely applicable, in particular to biochemical sample solutions such as blood serum.

**Figure 8:**
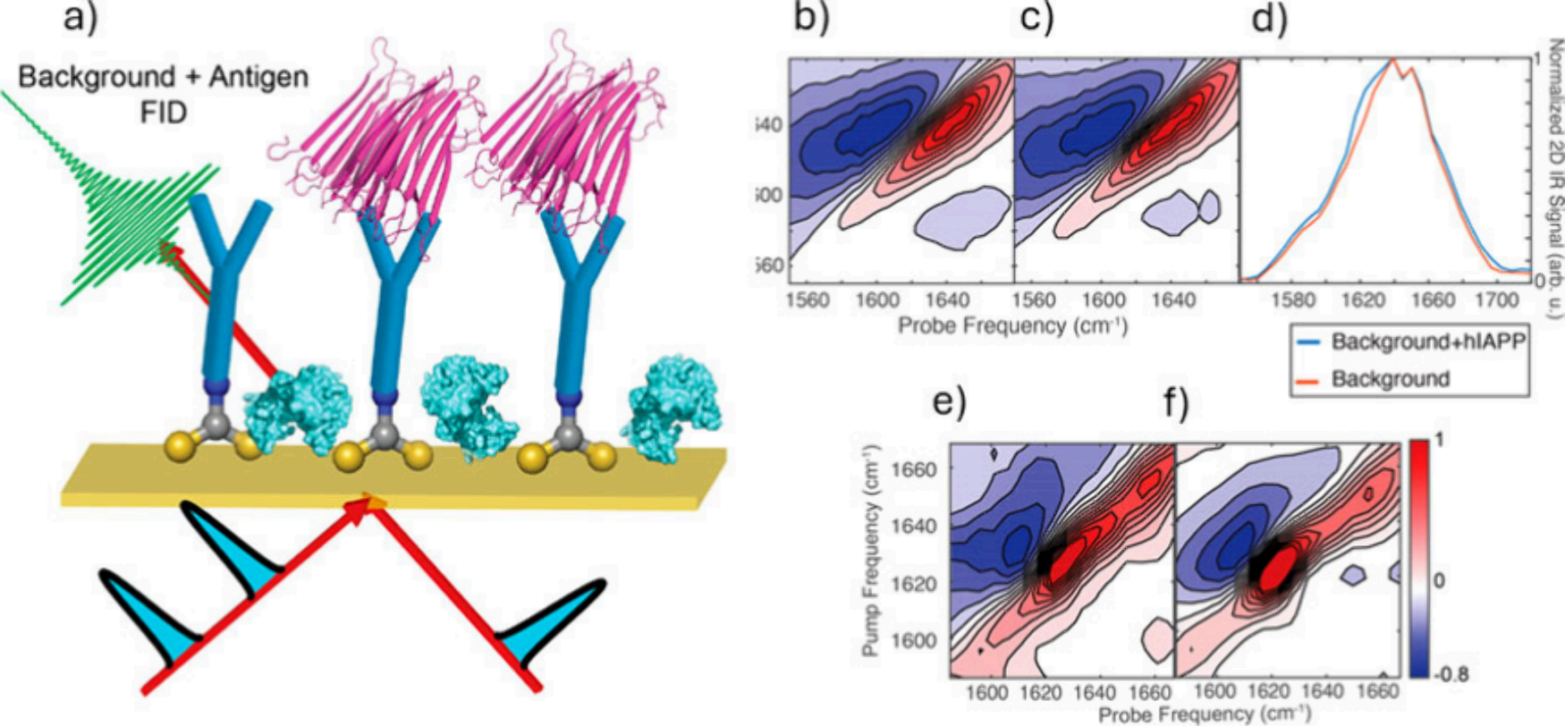
a) schematic diagram showing the 3 nm gold film covered with casein (to avoid nonspecific binding of IAPP to the gold) and IAPP bound to covalently attached polyclonal IAPP antibodies. Right: (b) 2DIR spectrum with IAPP; (c) 2DIR spectrum without IAPP; (d) normalized diagonal slices; (e) difference spectrum (b,c); (f) bulk 2D-IR spectrum of the same IAPP amyloids. Figures adapted from ref 89. Copyright 2019 American Chemical Society.

### 5.4 Improved Noise-Reduction and Scatter-Suppression Methods

Many interesting samples, such as fibrils, porous materials, and pressed pellets, scatter part of the infrared pump light into the infrared detector. This scattered light gives rise to two types of unwanted contributions to the observed light intensity: (i) heterodyne contributions due to interference of the scattered-pump *E*-field with the local-oscillator and third order signal *E*-field (corresponding to cross terms in the expression for the total observed light intensity |*E*_total_|^2^), and (ii) an intensity contribution due the added scattered-pump intensity (corresponding to an |*E*_pump_|^2^ term). The heterodyne contributions, which for small scatter intensity are dominant, can be completely eliminated by phase cycling.^188^ To also eliminate the intensity contribution from the observed signal, one can measure the observed light intensity both in the presence and absence of the probe/local-oscillator pulse using an optical chopper and subtract the two signals. In theory (and assuming negligible pulse-to-pulse intensity fluctuations), this should work perfectly. However, generally the observed intensities with and without probe/local-oscillator pulse differ strongly, and since HgCdTe (MCT) detectors have a very nonlinear response to the light intensity, this causes the probe-chopping approach to break down. To circumvent this issue, Ge et al.^189^ use a method that is commonly used in pump–probe spectroscopy: instead of chopping the probe beam, one measures the signal at sufficiently large negative *T*_w_, where the third-order signal is zero, but the scattered pump intensity remains the same, and subtracts the two signals. This makes it possible to measure 2D-IR spectra of samples for which the scatter is 20 times larger than the third-order signal. One might think that simply correcting for the nonlinearity of the MCT by calibrating it (recording a voltage vs intensity curve for each pixel) should also solve the problem, but Ge et al. show that in practice this does not work as well as negative-delay subtraction.^189^

In general, 2D-IR signals are measured using optical chopping and/or phase cycling and measuring and comparing the local-oscillator intensity (more precisely, adding or subtracting its logarithm) over several subsequent pulses. As a consequence, shot-to-shot fluctuations of the IR pulses become an important (and often the main) source of noise. The traditional way of correcting for these fluctuations (and the resulting baseline noise) is by using a beam splitter and referencing, i.e. simultaneously measuring the local-oscillator pulse intensity and the intensity of a copy of the local-oscillator pulse (the ‘reference’) that does not interact with the pump pulse(s). However, this requires a dual-row array detector (which is more expensive than a single-row one), and careful alignment of the probe and reference beams. Robben and Cheatum^190^ devised a much simpler method to correct for pulse-to-pulse fluctuations. To estimate the pulse-to-pulse intensity fluctuations, they use the readout of a subset of pixels in the array located at frequencies where the 2D-IR signal is zero (Figure 9). The pulse-to-pulse deviations of these readouts from their time-average values are then used to estimate the intensity-fluctuation-induced deviations of all the remaining pixels. The most basic case of this scenario would be to use the readout of the two pixels at the outer edges of the array (provided the 2D-IR signal is zero there) and use linear interpolation to estimate the shot-to-shot deviations of all the pixels in between. But inspired by a study of Feng et al.,^191^ Robben and Cheatum^190^ use a powerful generalization of this idea: a large set of pixels is used for referencing (basically all pixels where one can be sure the 2D-IR signal is zero can be used), and the readouts of these pixels are used to estimate the shot-to-shot deviations of all the other pixels in the detector array, assuming a generalized linear relation between the deviations. Specifically, if the shot-to-shot deviations of the reference pixels are put in a vector ***n***_ref_ (one entry for each reference pixel, and one such vector for each laser shot), and the deviations of the complete array in a vector ***n***_sig_, it is assumed that ***n***_sig_ = ***B.n***_ref_, where the correlation matrix ***B*** can be determined simply by blocking the pump pulse, measuring a sufficient number of instances of the vectors ***n***_ref_ and ***n***_sig_ and using a least-squares fit to optimize the matrix ***B***.^190^ This idea is especially elegant because it only requires a simple modification of the measurement software.

**Figure 9:**
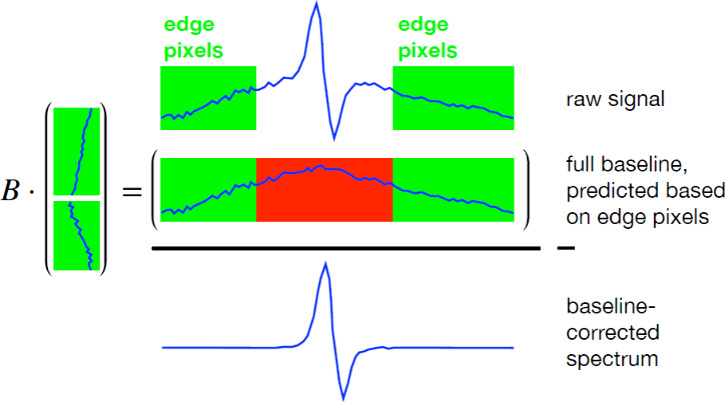
Correcting for baseline fluctuations using the edge pixels of an array detector. Left top: full spectrum as measured by the array detector. Left bottom: those parts of the total spectrum in which the 2D-IR signal is known to be zero are used to predict the deviation of the baseline from its time-average using a correlation matrix B which is determined from a separately recorded set of blank spectra. Right: The predicted baseline deviation is subtracted from the total spectrum to obtain the baseline-corrected spectrum.

## 6 Combining 2D-IR with Machine Learning

As discussed above, the high information density of 2D-IR spectra, along with the ability to measure large data sets, invites the extension of data analysis methods to include such tools as machine learning, which function effectively as a means to pattern recognition. Here we review studies which have begun to assess the suitability of this combination for analytical applications.

### 6.1 Protein-2D-IR Analysis Using Machine Learning

Due to the spectral congestion already mentioned earlier, it seems difficult if not impossible to obtain protein conformations directly from 2D-IR spectra in the same way as they can be obtained from 2D-NMR spectra. Exciton-based models can be used to calculate 2D-IR spectra based on a known protein conformation, often giving good agreement with the experimentally observed 2D-IR spectrum, and demonstrating its conformation sensitivity. But the 2D-IR spectra of proteins are generally regarded as fingerprints, the shape of which is determined by the conformation, but which cannot be directly ‘inverted’ to obtain the full protein conformation. Recently it has become clear that machine-learning based methods are well suited for making optimal use of the conformation-sensitivity of 2D-IR.

Probably the most basic characterization of protein conformation is its secondary-structure content: it is often related to protein functionality (e.g., transmembrane proteins have high α-helix content) and can be used to check whether the protein is in its native conformation (and not e.g. aggregated into amyloids which have high β-sheet content). Hence, knowing the secondary-structure content is extremely useful, in particular when a high-resolution structure is not available. Currently, the secondary structure content is often determined using UV circular dichroism, but this is not always reliable. Since α-helices and β-sheets have such distinct 2D-IR spectral signatures, 2D-IR spectroscopy may be a very reliable way to obtain the secondary-structure content of proteins. As described above Baiz et al.^41^ obtained promising results (∼ ± 9% error) using singular-value decomposition of the 2D-IR spectra of 16 proteins with varying secondary-structure content measured in D_2_O. Building on this, a recent machine-learning based approach was used to estimate secondary-structure content from 2D-IR spectra, using the support-vector machine (SVM) for classification and for regression, and a larger set of proteins and 2D-IR spectra, measured in H_2_O.^40^ The SVM for classification could confidently classify proteins as α-helix, β-sheet, or “mixed”, and the SVM for regression provided estimates of the α helix and beta-sheet content values with root-mean-square errors below 7% with respect to the values obtained from the X-ray structure. In this study it was again found that the 2D-IR spectrum provides more accurate estimates than the conventional FT-IR spectrum. Closer analysis of the results made it possible to identify which specific part of the 2D-IR spectrum had the highest statistical significance, and interestingly, this turned out to be a region located roughly between the diagonal and the high-pump-frequency β-sheet cross peak (Figure 10). Since the 2D-IR signal in this region is a complicated overlap of the α-helix and β-sheet diagonal peaks and the high-frequency β-sheet cross peak, it is indeed very sensitive to the secondary structure content (Figure 10), but it seems not very likely that any (human) spectroscopist would have chosen it as a marker, providing a nice demonstration of the added value of the machine-learning approach.

**Figure 10:**
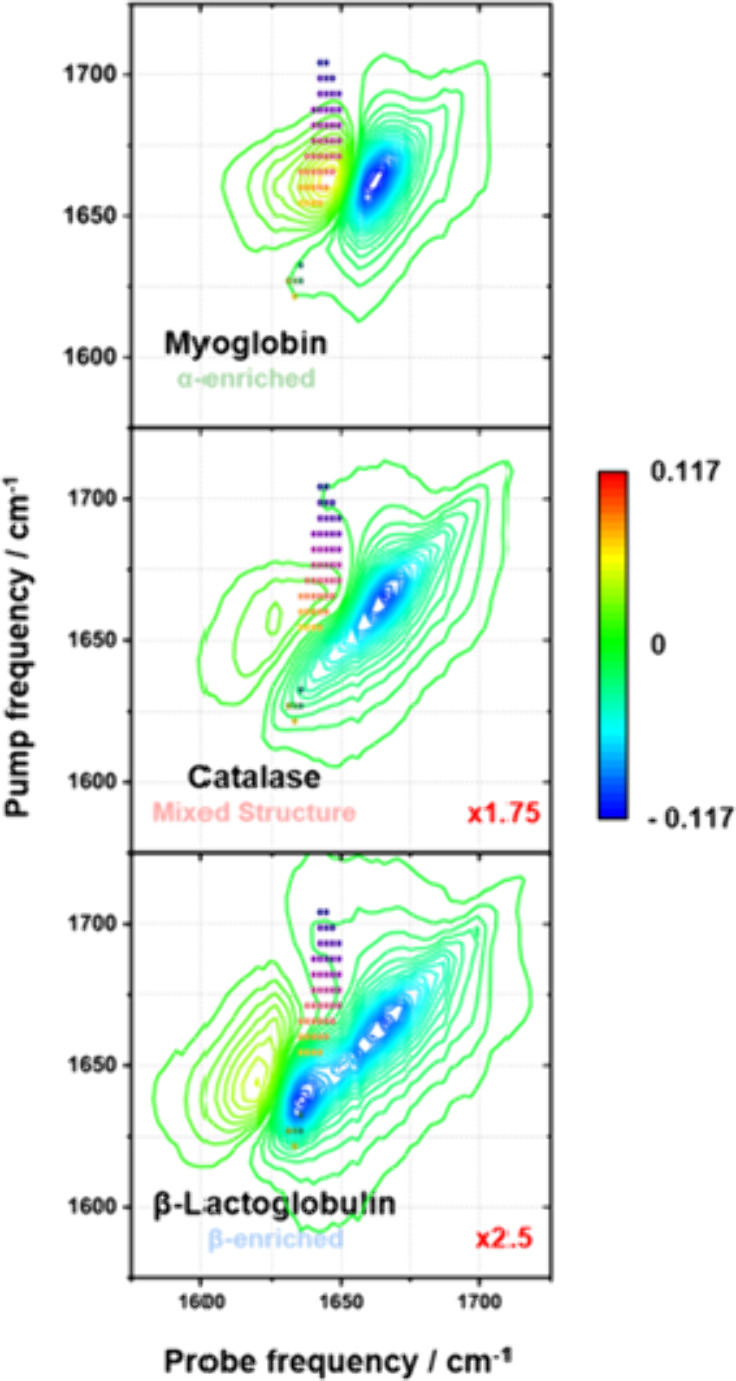
Machine learning can be used to estimate the secondary structure content of a protein from its 2D-IR spectrum. These three proteins differ in secondary-structure content, but although their 2D-IR spectra are different, it is not easy to translate the 2D-IR spectra into secondary-structure fractions, whereas a support-vector machine approach can do this reliably. The small rectangles indicate those parts of the 2D-IR spectrum that are most significant for structure prediction (viz. that have the highest F-values from an ANOVA-F test). Adapted from ref 40. Available under a CC-BY-3.0 license. Copyright Neil T. Hunt.

On a different note, Cheatum et al.^161^ demonstrated that machine learning classification could be used to identify subtle changes in the 2D-IR line shape of MeSCN solutions on the basis of their solvent. This study introduced the use of neural networks with 2D-IR data, which effectively treat spectra as images.

Returning to proteins, amide-I 2D-IR spectra contain contributions from many modes (the number of excitonic amide-I modes equals the number of residues, although some might have low IR cross section and hence be invisible), and given the homogeneous line width of about 10 cm^–1^ and the total spectral range of about 80 cm^–1^ in which all the amide I modes are contained, the spectral congestion is inevitably very strong, so it seems practically impossible to obtain the absolute, atomistic conformation of a protein from a 2D-IR spectrum alone. However, a recent study has reported a machine-learning approach in which the information on the amide-I 2D-IR spectrum is used in combination with an AI-based folding algorithm to derive the full conformation of a wide variety of proteins.^164^ In this study, the machine-learning protocol predicts a matrix containing all C**α**–C**α** distances (the ‘distance map’) from a 2D-IR spectrum. Interestingly, the ‘2D-IR fingerprint’ metaphor in this case becomes quite literal, in that the algorithm used to extract features from the 2D-IR spectra (Google’s DeepLabV3)^164^ was specifically designed for image analysis. The predicted distance map (containing some *n*(*n*-1)/2 distances, with *n* the number of C**α** atoms) is then used as input for a gradient-based folding algorithm to obtain the protein conformation. The results are impressive, but it should be noted that the training data set consisted completely of calculated 2D-IR spectra (based on the PDB structure) with a very small homogeneous line width and no inhomogeneous broadening, which renders these spectra somewhat unrealistically rich in structure. The authors point out that the use of calculated spectra was inevitable because of the lack of experimental 2D-IR spectra, an issue that we will discuss below.

### 6.2 Blood Serum Analysis

Two other successful applications of machine-learning-assisted 2D-IR, the detection and characterization of protein–drug binding in blood serum,^128^ and the classification of blood serum samples from patients with melanoma,^148^ were already discussed in detail above in relation to biomedical analysis. Such samples pose a different problem to the secondary structure evaluation and structural recovery applications discussed above because, while there is a well-defined theoretical relationship between protein structure and the 2D-IR response that can, in principle, be learned by an algorithm given enough data, a blood serum sample has many more variables to contend with, not least the fact that blood proteins vary in concentration from one person to the next and from one moment to the next depending on metabolic processes and daily rhythms. This means that, while one can envisage a single large 2D-IR data library of proteins being amassed and used to solve the protein structure problem, in the biomedical context each study will have its own parameters, for example relating to the impact of treatments that the patients receive. As such each study would require its own data library collection, curation and algorithm development process. Despite this, machine learning will be invaluable as human evaluation of 2D-IR spectra of serum samples has been shown to be extremely challenging.^148^ The relative paucity of biomedical 2D-IR data means that it is likely that 2D-IR applications may be best deployed as part of a larger multidisciplinary study when targeting a specific disease or condition, for example by identifying groups of patients with similar responses to help with targeting a parallel proteomics study.

One area where machine learning-backed 2D-IR certainly has the potential be of considerable benefit would be in understanding the ‘healthy patient’ serum sample. The ability to measure protein fingerprints that encompass the ‘normal’ range of protein profiles and so to identify samples which fall outside of those ranges and are worthy of more investigation could be achieved by exploiting the speed and noninvasive simplicity of the 2D-IR test along with machine learning algorithm development. Once again, a concerted effort to build up the necessary database of samples would be required. Over time, monitoring of an individual patient could identify a sudden change in health, while such a measurement protocol would lead toward the kind of understanding of the individual patient required to underpin the move toward personalized medicine. An extension of this type of study would use libraries of protein spectra for individual blood serum components and machine learning algorithms to produce concentrations of key proteins in each sample, thus adding quantitative data to the classification outcome.

## 7 Open Challenges for 2D-IR

We hope that the above overview has shown that 2D-IR spectroscopy has strong potential as a bioanalytical tool and have summarized the applications to date in Table 1. Prior to discussing some of the open challenges that face 2D-IR, we will pause to consider how the method, in its current guise, compares to other commonly used techniques. In some cases, it is possible to draw direct comparisons but, in many instances, it is the complementarity of 2D-IR with established tools that is most apparent. For example, comparing 2D-IR with FT-IR highlights the improvements in spectral peak resolution and sensitivity that we have described above as well as the ability of 2D-IR to suppress solvent bands that impair FT-IR. A comparison with Raman spectroscopy is hard to achieve, because the naturally opposing strengths of Raman and IR methods are well-known to be complementary, with many vibrational bands appearing strongly in Raman and weakly in IR spectroscopy and *vice versa*. Similarly, Raman lends itself to surface and resonant enhancement in a way that IR and 2D-IR do not. In the case of CD spectroscopy, we have shown that 2D-IR can achieve at least comparable secondary structure quantification accuracy,^85^ but the instrumentation required for CD is cheaper and more accessible at the current time than 2D-IR spectroscopy. CD also tends to be far more sensitive owing to its use of electronic transitions with larger transition dipole moments.

**Table 1:** Summary of Bioanalytical Applications of 2D-IR

Section	Ref	Sample	Objective	Comments
2.2	^26^	[NiFe] hydrogenase enzyme	Spectroscopy and identification of redox states and	Organometallic carbonyl active sites; enzyme concentration: 270 μM; few mOD absorbance in FTIR
2.3/4.1	^32^	Mixture of Ir and Co dicarbonyl species	Mixture analysis and spectral separation	2 mM solution in hexane
3.1 and 3.2	^41^	Sixteen proteins	Secondary structure quantification	20 mg/mL in D_2_O
3.2	^85^	Calmodulin	Quantification of secondary structure change	0.8 mM in deuterated buffer solution
3.2	^87^	Antifreeze glycoproteins	Structure determination	2 wt % in D_2_O
3.2	^88^	Lysozyme and α-chymotrypsin	Structure change during denaturation	10–20 mg/mL in D_2_O
3.2/5.3	^89^	Antibody-tethered human islet amyloid polypeptide	Fibril morphological analysis	Monolayer sensitivity using ATR geometry 2D-IR measurement exploiting surface enhancement from Au surface
3.2	^93^	Ten proteins	Protein differentiation based on peptide spectra	EVV 2D-IR spectroscopy; differentiation achieved in 4–5 min per protein; samples: dried films cast onto glass slides; concentrations 10–50 mg/mL; 1.5 μL deposited
3.3	^94^	Human islet amyloid polypeptide	Fibril-formation kinetics	Automated 2D-IR spectroscopy, 1 min per sample. Concentration 1 mM
3.3	^95^	Islet amyloid polypeptide	Effect of membrane on amyloid formation	Large unilamellar vesicles formed from 80 mM lipid concentration in D_2_O
3.3	^96^	Islet amyloid polypeptide	Amyloid inhibition	Concentration: 250 μM in the presence and absence of acid fuchsin
3.3	^98^	Apolipoprotein A-I	Detection of amyloid formation	Solution and solid phase data collection
3.3	^107^	α-Synuclein	Fibril structure	Monomer concentration: 150 μM
3.3	^108^	α-Synuclein	Impact of N-terminal acetylation membrane binding and fibril structure	Deuterated PBS buffer at 37 °C
3.3	^106^	α-Synuclein	Fibril structure after C-terminal truncation	150 μM; D_2_O; 15 μL sample volume
3.3	^99^	Islet amyloid polypeptide	Identification of intermediate structures	40 mM IAPP concentration
3.3	^84^	Islet amyloid polypeptide	Identification of fibril structural differences caused by micelles, membranes, solution	1–5 mM concentration, D_2_O, few μL sample volume
3.3	^100^	Crystallin proteins in porcine eye lens	Presence of amyloid aggregates in cataracts	Lens slices deposited directly onto a CaF_2_ window and deuterated with D_2_O
3.3	^102^	Crystallin proteins in human eye lens	Presence of amyloid aggregates in cataracts	Lens slices of 25 μm thickness deposited onto a CaF_2_ window. Tissues were dried under N_2_ then placed into a flow sample cell (56–100 μm length). Deuterated PBS was then added.
3.3	^112^	Human islet amyloid polypeptide	Fibril polymorph differentiation	Cross-peak specific polarization scheme used to differentiate polymorphs
3.3	^113^	F-actin	Response of filament secondary structure to molecular crowding	Actin concentration: 1.5 mg/mL (36 μM) in deuterated buffer solution
3.3	^114^	Gramicidin-S	Aggregation of cyclic antibiotic peptide	30 μL volume of Gramicidin-S solution (5 mg/mL in 1-octanol)
3.3	^119^	Insulin	Characterization of the monomer–dimer transition	Protein dissolved in D_2_O at 1 mg mL
3.3	^127^	InhA protein	Impact of drug binding on protein structure and dynamics	Sample concentration range 20–30 mg/mL (0.8 mM) in deuterated buffer
3.3/3.4/4.2/6.2	^128^	Human blood serum	Detection of drug molecules via interactions with serum albumin	Pooled blood serum spiked with four common drugs at physiologically relevant levels; use of machine learning in analysis strategy
3.3	^129^	IL-17 cytokine	Dynamic impact of drug binding	20 μL aliquot of a solution of IL-17 (∼100 μM, in a deuterated buffer)
3.3	^130^	DNA oligomers and ligands	Screening of DNA-ligand complexes	Double stranded DNA concentration: 10 mM in deuterated buffer
3.3	^131^	FGFR kinase	Detection of drug binding	EVV-2DIR; samples: protein gel spots on CaF_2_ windows
3.3	^132–136^	Calmodulin and lanmodulin	Effects of ion binding	1–1.5 mM protein concentration
3.4/4.2	^25^	Blood serum	Detection of proteins in H_2_O-rich media	Blood serum, 3 μm path length
3.4	^105^	Insulin	amyloid aggregation dynamics in H_2_O	Concentration: 17 mg/mL (monomer), 1–2 mg/mL (fibril)
4.1	^152^	Sacubitril allisartan calcium	Determination of differences in structures in cocrystals	Crystal suspensions in CH_2_Cl_2_
4.1	^153^	Coffee/milk mixtures	Amide I spectroscopy of milk proteins in coffee	Samples diluted 10× with D_2_O
4.1	^154^	Silaffin peptide R5	Intermolecular interactions relevant to biomineralization	1–2.5 mM peptide in D_2_O and silica–R5 particle suspension
4.1	^23^	*Bacillus atrophaeus* and *Bacillus thuringiensis* spores	Spore detection and identification	Spores as dry films and in suspension
4.2	^144,146^	Blood serum	Detection of change in protein profile	Blood serum plus ‘spiked’ proteins, 3 μm path length
4.2/6.2	^148^	Blood serum	Detection of changes in clinical samples	Blood serum, 3 μm path length; Analysis used machine learning
4.2	^103^	Human islet amyloid polypeptide	Comparing fibril polymorph found in blood serum to that in solution	Blood serum, depleted of serum albumin and IgG proteins, dried and rehydrated with D_2_O
4.2	^18^	Mouse pancreatic tissue	Hyperspectral tissue imaging	Formalin fixed mouse pancreatic tissue sections
4.2	^91^	Mouse kidney	Spectral imaging of tissue	Protein CH_3_ and hematoxylin-based imaging of mouse kidney sections – 5 μm thick
5.1	^168^	Bovine serum albumin	Separation and detection of amyloids and monomers	2D-IR DOSY spectroscopy used to separate mixtures based on molecular size
6.1	^40^	Thirty-five proteins	Protein structure analysis	Machine-learning based classification and secondary structure quantification

The comparison with NMR is an interesting one based on the obvious relationships between the two experimental methods. In this case we can draw on the study highlighted above that combined 2D-IR and NMR measurements on the same protein (IL-17).^129^ 2D-IR measurements used less material, requiring 20 μL of sample at 100 μM concentration, while NMR required 350 μL at 75–200 μM depending on the protein. It is also the case that NMR required extensive isotopic labeling and enrichment while NMR experiments and their analysis took considerably longer than those for 2D-IR. Conversely, NMR was able to deliver atomistic structures and site-specific insight, while 2D-IR gave broader structural information more rapidly. Thus, we believe this is a good example of the natural complementarity of the methods, particularly when we take into account the sensitivity of the two methods to different dynamic time scales.

Mass spectrometry (MS) techniques are widely used for protein analysis, but comparisons are harder to draw. 2D-IR studies whole proteins in solution but at lower sensitivity, while MS tends to require digestion or transition to the gas phase meaning that the two methods answer very different questions.

Finally, we consider the gold standard structural methods of X-ray crystallography and cryo-electron (cryo-EM) microscopy. While 2D-IR spectroscopy cannot compete with the atomistic structural insight of these powerhouse methods and crystallography can now be done with very high throughput, albeit requiring preparation of crystalline samples, the ability to study proteins in their physiological environments and to observe their dynamics is a key ability that both solid phase structural tools lack. This is also true of the justifiably lauded AI-based structure prediction tools such as AlphaFold. While these can produce accurate structure predictions from just a primary amino acid sequence, they are trained on data obtained via crystallography and cryo-EM such that they too are insensitive to the dynamic behavior of proteins that can be crucial to function.^192–194^

Considering the bigger picture then, while it may not become a standard analytical tool, largely as a result of the complex technology involved, the above examples demonstrate that 2D-IR has unique features (structural information content, short acquisition time, no sample preparation needed) that, in specific cases, make it the best available bioanalytical method and so there is potential for it to exist as a valuable member of our family of analytical methods.

In this last section, we move on to discuss a number of remaining open challenges.

### 7.1 Compatibility with Separation Methods such as HPLC

Much of traditional analytical chemistry is separation science, i.e. determining the composition of complex mixed samples, such as blood serum and other biological fluids. Typically, chromatography methods are used for this purpose, and the detection at the output of the chromatographic column peaks is done with a wide variety of detection methods, ranging from UV absorption or fluorescence to mass spectroscopy, that have varying degrees of structure sensitivity.^195^ Hyphenating a chromatography column with a 2D-IR spectrometer would make it possible to detect compounds and simultaneously provide structural information that might be difficult to obtain with other methods. At present, the sensitivity of 2D-IR is insufficient to realize such a combination, making this a longer-term goal. The surface enhancement discussed above strongly increases the sensitivity of 2D-IR spectroscopy, but as the amplification effect occurs only at very short distances from the gold surface, it seems difficult to apply this enhancement in a flow setup, so this challenge remains to be addressed.

### 7.2 Creating a FAIR Online 2D-IR Database

As we saw in the previous section, machine learning methods are a promising new approach to addressing the problem of spectral congestion in 2D-IR spectroscopy. But all machine learning methods need data sets (the bigger the better), and as was already noted, there is currently hardly any experimental 2D-IR data available online. As a consequence, current studies of machine-learning assisted 2D-IR have to create their training data sets themselves, either by simulations or experiments. For this new 2D-IR-research field to blossom it is crucial that as much 2D-IR data as possible is shared online; and importantly, that it is made available in accordance with the FAIR principles (Findable, Accessible, Interoperable, Reproducible).^196^ We believe that this could be a shorter-term goal and hope this review will inspire efforts to set up an online database of FAIR protein-2D-IR spectra. This would be valuable for machine-learning 2D-IR studies, but such a database would also make it possible to assess how reproducible 2D-IR spectra are from lab to lab.

The wide variation in instruments currently in use within the community, which span different laser types, powers, repetition rates and bandwidths as well as measurement geometries and sample formats will make standardization challenging. However, such quantitative characterization of lab-to-lab reproducibility is standard practice in analytical chemistry and so this is a necessary next step if 2D-IR is to continue its current rate of progress. It is plausible that these problems may be addressable using data science approaches or the introduction of a community standard set of measurements to allow cross-comparisons of data sets. This is an open challenge for the community to address but, if it can be achieved, an online protein-2D-IR database would enhance new developments in 2D-IR data analysis in a manner akin to the Protein Data Bank and could also help to make 2D-IR a more robust and widely used bioanalytical tool.

### 7.3 Toward an Accessible Spectroscopy Instrument

The work reviewed here and the open challenges discussed highlight considerable potential for 2D-IR to contribute positively as part of a suite of (bio)­analytical tools and there are clearly areas in which 2D-IR fills a gap in our current capability. A significant barrier to progress lies in the current instrumental state-of-the-art. While high pulse repetition rate lasers and solid-state pulse shaping spectrometers represent a considerable advance over the spectrometers of 20 years ago, the 2D-IR spectroscopist still needs considerable specialist skills to operate the equipment, collect and analyze the data. Thus, in the short-to-medium term 2D-IR is likely to operate via a service facility model. To achieve its potential, 2D-IR needs to progress to a largely hands-free instrument with standardized measurement and data processing protocols. The former is perhaps a longer-term challenge for the instrument manufacturers, but there is growing evidence of the demand that might turn into a viable market. The latter is a challenge for the 2D-IR community that goes hand-in-hand with the previous two challenges. If achieved together, it is possible that 2D-IR spectroscopy could break out of the laser laboratory and into pharmaceutical companies, hospital laboratories and even factories.
